# Supplementary material for: Self-help and mutual assistance in the aftermath of a tsunami: How individual factors contribute to resolving difficulties
Source: PLoS One. 2021 Oct 7;16(10):e0258325. doi: 10.1371/journal.pone.0258325 (PMC8496872; doi:10.1371/journal.pone.0258325)
Supplement: S1 Results — Including S1-S16 Tables. (DOCX) [file pone.0258325.s001.docx]

Self-help and mutual assistance in the aftermath of a tsunami: how individual factors contribute to resolving difficulties

Motoaki Sugiura ^1, 2, 3,^ *, Ryo Ishibashi ^2, 3^ , Tsuneyuki Abe ^4^ , Rui Nouchi ^2, 3^ , Akio Honda ^5^ , Shosuke Sato ^1^ , Toshiaki Muramoto ^1^ and Fumihiko Imamura ^1^

^1^ International Research Institute of Disaster Science, Tohoku University, Sendai, Japan

^2^ Institute of Development, Aging and Cancer, Tohoku University, Sendai, Japan

^3^ Smart-Aging Research Center, Tohoku University, Sendai, Japan

^4^ Graduate School of Arts and Letters, Tohoku University, Sendai, Japan

^5^ Faculty of Informatics, Shizuoka Institute of Science and Technology, Fukuroi, Japan

***** Correspondence: sugiura@tohoku.ac.jp (M.S.)

**Supplementary results**

**S1 Table.** **Experiences of difficulty and how they were resolved**.

**S2 Table.** **Personality factors.**

**S3 Table.** **Exacerbating factors for general life difficulty.**

**S4 Table.** **Exacerbating factors for medico-psychological difficulty.**

**S5 Table.** **Facilitative factors for resolving general life difficulty by oneself.**

**S6 Table.** **Facilitative factors for resolving medico-psychological difficulty by oneself.**

**S7 Table.** **Facilitative factors for resolving general life difficulties by requesting help.**

**S8 Table.** **Facilitative factors for resolving medico-psychological difficulties by requesting help.**

**S9 Table.** **Facilitative factors for resolving** **general life difficulties through family.**

**S10 Table.** **Facilitative factors for resolving** **medico-psychological difficulties through family.**

**S11 Table.** **Facilitative factors for resolving** **general life difficulties through the help of an acquaintance.**

**S12 Table.** **Facilitative factors for resolving** **medico-psychological difficulties through the help of an acquaintance.**

**S13 Table.** **Facilitative factors for resolving** **general life difficulties through cooperation.**

**S14 Table.** **Facilitative factors for resolving medico-psychological difficulties through cooperation.**

**S15 Table.** **Facilitative factors for resolving** **general life difficulties through public assistance.**

**S16 Table.** **Facilitative factors for resolving** **medico-psychological difficulties through public assistance.**

**S1 Table.** **Experiences of difficulty and how they were resolved**.

| **Trouble Item** | | **Difficulty** | | | **Solution** | | | | | | |
| --- | --- | --- | --- | --- | --- | --- | --- | --- | --- | --- | --- |
|  |  | 0 | 1 | 2 | S | R | F | A | C | P | U |
| 1. | Eating | 252 (19%) | 599 (45%) | 495 (37%) | 289 (25%) | 30 (3%) | 563 (49%) | 380 (33%) | 274 (24%) | 507 (44%) | 121 (10%) |
| 2. | Cooking | 275 (21%) | 441 (34%) | 584 (45%) | 357 (31%) | 11 (1%) | 454 (40%) | 197 (17%) | 145 (13%) | 233 (20%) | 237 (21%) |
| 3. | Appropriate spare clothes | 489 (37%) | 374 (28%) | 465 (35%) | 246 (27%) | 30 (3%) | 346 (37%) | 225 (24%) | 62 (7%) | 259 (28%) | 178 (19%) |
| 4. | Room temperature | 277 (21%) | 449 (34%) | 589 (45%) | 397 (35%) | 16 (1%) | 361 (32%) | 148 (13%) | 134 (12%) | 161 (14%) | 275 (24%) |
| 5. | Sleeping | 455 (34%) | 470 (35%) | 400 (30%) | 343 (36%) | 3 (0%) | 220 (23%) | 43 (4%) | 116 (12%) | 70 (7%) | 323 (34%) |
| 6. | Access to a toilet | 380 (29%) | 406 (30%) | 546 (41%) | 401 (39%) | 9 (1%) | 236 (23%) | 72 (7%) | 167 (16%) | 201 (19%) | 207 (20%) |
| 7. | Washing one’s face | 340 (26%) | 463 (35%) | 520 (39%) | 418 (39%) | 6 (1%) | 276 (26%) | 87 (8%) | 111 (10%) | 166 (15%) | 263 (25%) |
| 8. | Bathing | 109 (8%) | 283 (21%) | 947 (71%) | 229 (18%) | 25 (2%) | 474 (36%) | 218 (17%) | 53 (4%) | 270 (21%) | 366 (28%) |
| 9. | Laundry | 156 (12%) | 338 (26%) | 829 (63%) | 389 (31%) | 24 (2%) | 451 (36%) | 131 (10%) | 57 (5%) | 87 (7%) | 374 (30%) |
| 10. | Information gathering | 243 (18%) | 479 (36%) | 595 (45%) | 268 (23%) | 63 (5%) | 311 (27%) | 226 (19%) | 248 (21%) | 268 (23%) | 307 (26%) |
| 11. | Transportation | 267 (20%) | 364 (28%) | 689 (52%) | 404 (35%) | 44 (4%) | 382 (33%) | 177 (15%) | 63 (6%) | 69 (6%) | 320 (28%) |
| 12. | Medical care for oneself | 808 (62%) | 285 (22%) | 216 (17%) | 144 (24%) | 20 (3%) | 115 (19%) | 19 (3%) | 17 (3%) | 135 (22%) | 228 (38%) |
| 13. | Medical care for one’s family | 751 (59%) | 274 (22%) | 243 (19%) | 121 (18%) | 27 (4%) | 187 (28%) | 39 (6%) | 28 (4%) | 144 (22%) | 239 (36%) |
| 14. | Psychological stress | 569 (43%) | 462 (35%) | 284 (22%) | 303 (36%) | 11 (1%) | 271 (32%) | 105 (12%) | 61 (7%) | 54 (6%) | 277 (33%) |
| 15. | Psychological care for one’s family | 569 (44%) | 465 (36%) | 259 (20%) | 218 (26%) | 22 (3%) | 331 (39%) | 100 (12%) | 68 (8%) | 58 (7%) | 288 (34%) |
| 16. | Noise | 889 (68%) | 257 (20%) | 153 (12%) | 112 (21%) | 3 (1%) | 43 (8%) | 12 (2%) | 67 (13%) | 34 (7%) | 300 (57%) |
| 17. | Stench | 573 (44%) | 369 (28%) | 367 (28%) | 176 (21%) | 9 (1%) | 71 (8%) | 31 (4%) | 85 (10%) | 144 (17%) | 462 (55%) |
| 18. | Privacy | 664 (51%) | 330 (25%) | 316 (24%) | 230 (31%) | 4 (1%) | 127 (17%) | 35 (5%) | 105 (14%) | 47 (6%) | 340 (45%) |

For each item of difficulty, frequency data (effective %) for three levels of difficulty experienced (0: no, 1: a little, or 2: very much) and seven types of solutions are given. Solution types are S: Self (by one’s own effort), R: Request (asking someone for help), F: Family (the help of family members or relatives), A: Acquaintances (the help of acquaintances), C: Cooperation (mutual cooperation among refugees), P: Public (public support, such as municipality, military, or volunteers), and U: Unsolved (not solved).

**S2 Table.** **Personality factors.**

| **Factor** | **Example item** | **Score** |
| --- | --- | --- |
| **Power to Live** | | |
| Leadership | *I gather together everyone involved to discuss how to resolve a problem.* | 51 ± 19 |
| Problem solving | *When unsure of what I should do, I compare several alternative actions.* | 66 ± 15 |
| Altruism | *I like it when other people rely on me and are grateful to me.* | 63 ± 16 |
| Stubbornness | *I am stubborn and always get my own way.* | 60 ± 17 |
| Etiquette | *On a daily basis, I take the initiative to greet family members and people in the neighborhood.* | 82 ± 15 |
| Emotional regulation | *I endeavor not to brood during difficult times.* | 66 ± 16 |
| Self-transcendence | *I am aware that I am alive and have a sense of responsibility while living.* | 71 ± 16 |
| Active well-being | *In everyday life, I have habitual practices that are essential for relieving stress or giving me a change of pace.* | 58 ± 21 |
| **Big Five dimensions** | | |
| Extraversion | *I think I am enthusiastic and extraverted.* | 51 ± 21 |
| Agreeableness | *I think I am sympathetic and warm.* | 70 ± 17 |
| Conscientiousness | *I think I am dependable and self-disciplined.* | 56 ± 20 |
| Neuroticism | *I think I am anxious and easily upset.* | 50 ± 19 |
| Openness | *I think I like new things and have unusual ideas.* | 48 ± 21 |

For each factor or dimension, an example of the question (italics; originally in Japanese) and mean ± standard deviation (SD) of the total score (ration in percentile) are given.

**S3 Table.** **Exacerbating factors for general life difficulty.**

| **Background factors** | | | | | | | | | | | |
| --- | --- | --- | --- | --- | --- | --- | --- | --- | --- | --- | --- |
|  | Block 1 (damage) | | | | + Block 2 (demographic) | | | | | | |
|  | β | std β | *ｔ* | | β | std β | *ｔ* | |  |  |  |
| Household goods | 0.269 | 0.118 | 3.977 | ** | 0.240 | 0.105 | 3.554 | ** |  |  |  |
| Car | 0.651 | 0.132 | 4.440 | ** | 0.750 | 0.152 | 5.092 | ** |  |  |  |
| Family injury | 0.909 | 0.072 | 2.492 | * | 0.851 | 0.067 | 2.352 | * |  |  |  |
| Death of a friend | 0.370 | 0.075 | 2.600 | * | 0.380 | 0.077 | 2.682 | * |  |  |  |
| Sex |  |  |  |  | -0.334 | -0.069 | -2.402 | * |  |  |  |
| Single |  |  |  |  | -0.914 | -0.097 | -3.364 | ** |  |  |  |
| Couple |  |  |  |  | -0.425 | -0.070 | -2.415 | * |  |  |  |
|  | *R^2^* | adj *R^2^* | *F*(4, 1151) | | *R^2^* | adj *R^2^* | *F*(7, 1148) | | *R^2^* change | *F* change | |
|  | 0.056 | 0.053 | 17.165 | ** | 0.074 | 0.069 | 13.146 | ** | 0.018 | 7.405 | ** |
| **Personality factors** (Block 3; one by one) | | | | | | | | | | | |
| *Not significant* | | | | | | | | | | | |

The results of the hierarchical regression analysis for the degree of difficulty experienced are given as β, standardized (std) β, *t*-value for each variable, *R^2^*, adjusted (adj) *R^2^*, and *F*- value (degree of freedom 1, 2) separately for each block. For background factors (i.e., blocks 1 and 2), variables were initially selected using a stepwise method (forward selection: *p* < 0.05, backward selection: *p* > 0.10) with limited samples and, for personality factors (i.e., block 3), those with significant contributions at an uncorrected level are shown. *: *p* < 0.05, uncorrected; **: *p* < 0.05 after correction for the multiple comparisons of the 31 variables for background factors and 13 variables for personality factors.

**S4 Table.** **Exacerbating factors for medico-psychological difficulty.**

| **Background factors** | | | | | | | | | | | |
| --- | --- | --- | --- | --- | --- | --- | --- | --- | --- | --- | --- |
|  | Block 1 (damage) | | | | + Block 2 (demographic) | | | | | | |
|  | β | std β | *ｔ* | | β | std β | *ｔ* | |  |  |  |
| Household goods | 0.816 | 0.296 | 8.919 | ** | 0.822 | 0.298 | 9.032 | ** |  |  |  |
| Car | 0.438 | 0.073 | 2.547 | * | 0.368 | 0.061 | 2.142 | * |  |  |  |
| Injury to oneself | 0.793 | 0.109 | 3.554 | ** | 0.780 | 0.107 | 3.512 | ** |  |  |  |
| Family injury | 1.707 | 0.112 | 3.715 | ** | 1.745 | 0.115 | 3.820 | ** |  |  |  |
| Death of a family member | 0.770 | 0.073 | 2.641 | * | 0.792 | 0.075 | 2.732 | * |  |  |  |
| Death of a friend | 0.546 | 0.091 | 3.295 | ** | 0.522 | 0.087 | 3.161 | * |  |  |  |
| Refugee life | 0.487 | 0.079 | 2.462 | * | 0.472 | 0.076 | 2.391 | * |  |  |  |
| Worker |  |  |  |  | -0.516 | -0.088 | -3.232 | ** |  |  |  |
| Household income |  |  |  |  | -0.099 | -0.056 | -2.045 | * |  |  |  |
|  | *R^2^* | adj *R^2^* | *F*(7, 1044) | | *R^2^* | adj *R^2^* | *F*(9, 1042) | | *R^2^* change | *F* change | |
|  | 0.220 | 0.214 | 41.961 | ** | 0.231 | 0.224 | 34.699 | ** | 0.011 | 7.463 | ** |
| **Personality factors** (Block 3; one by one) | | | | | | | | | | | |
| *Power to live* | β | std β | *ｔ* | | *R^2^* | adj *R^2^* | *F*(10, 971) | | *R^2^* change | *F* change | |
| Problem solving | -1.257 | -0.066 | -2.348 | * | 0.244 | 0.236 | 31.354 | ** | 0.004 | 5.514 | * |
| Emotional regulation | -1.273 | -0.071 | -2.521 | * | 0.245 | 0.237 | 31.465 | ** | 0.005 | 6.354 | * |
| Self-transcendence | -1.189 | -0.063 | -2.241 | * | 0.244 | 0.236 | 31.290 | ** | 0.004 | 5.024 | * |
| *Big 5* | β | std β | *ｔ* |  | *R^2^* | adj *R^2^* | *F*(10, 974) | | *R^2^* change | *F* change | |
| Extraversion | -1.322 | -0.095 | -3.354 | ** | 0.243 | 0.235 | 31.281 | ** | 0.009 | 11.246 | ** |
| Neuroticism | 1.628 | 0.108 | 3.843 | ** | 0.246 | 0.238 | 31.741 | ** | 0.011 | 14.766 | ** |

Details are the same as for S3 Table.

**S5 Table.** **Facilitative factors for resolving general life difficulty by oneself.**

| **Background factors** | | | | | | | | | | | |
| --- | --- | --- | --- | --- | --- | --- | --- | --- | --- | --- | --- |
|  | Block 1 (damage) | | | | + Block 2 (demographic) | | | | | | |
|  | β | std β | *ｔ* | | β | std β | *ｔ* | |  |  |  |
| Household goods | -0.056 | -0.183 | -5.326 | ** |  |  |  |  |  |  |  |
| Car | -0.067 | -0.105 | -3.469 | ** |  |  |  |  |  |  |  |
| Refugee life | -0.131 | -0.197 | -5.863 | ** |  |  |  |  |  |  |  |
|  | *R^2^* | adj *R^2^* | *F*(3, 1000) | |  |  |  |  |  |  |  |
|  | 0.130 | 0.127 | 49.632 | ** |  |  |  |  |  |  |  |
| **Personality factors** (Block 3; one by one) | | | | | | | | | | | |
| *Power to live* | β | std β | *ｔ* | | *R^2^* | adj *R^2^* | *F*(4, 927) | | *R^2^* change | *F* change | |
| Problem solving | 0.221 | 0.108 | 3.563 | ** | 0.152 | 0.148 | 41.554 | ** | 0.012 | 12.692 | ** |
| Emotional regulation | 0.164 | 0.084 | 2.777 | * | 0.148 | 0.144 | 40.107 | ** | 0.007 | 7.714 | * |
| Self-transcendence | 0.124 | 0.061 | 2.006 | * | 0.144 | 0.140 | 39.034 | ** | 0.004 | 4.025 | * |
| *Big 5* | β | std β | *ｔ* | | *R^2^* | adj *R^2^* | *F*(4, 935) | | *R^2^* change | *F* change | |
| Neuroticism | -0.124 | -0.074 | -2.434 | * | 0.140 | 0.136 | 37.961 | ** | 0.005 | 5.923 | * |

The results of the hierarchical regression analysis for the degree of resolution are given. **: *p* < 0.05 after correction for multiple comparisons of the 32 background factor variables and 13 personality factor variables. The degree of difficulty experienced was entered as an additional damage factor in the stepwise variable selection. Other details are the same as for S3 Table.

**S6 Table.** **Facilitative factors for resolving medico-psychological difficulty by oneself.**

| **Background factors** | | | | | | | | | | | |
| --- | --- | --- | --- | --- | --- | --- | --- | --- | --- | --- | --- |
|  | Block 1 (damage) | | | | + Block 2 (demographic) | | | | | | |
|  | β | std β | *ｔ* | | β | std β | *ｔ* | |  |  |  |
| Car | -0.039 | -0.054 | -1.705 |  | -0.044 | -0.061 | -1.910 |  |  |  |  |
| Refugee life | -0.119 | -0.154 | -4.826 | ** | -0.110 | -0.142 | -4.453 | ** |  |  |  |
| Difficulty experienced | -0.008 | -0.057 | -1.776 |  | -0.008 | -0.062 | -1.934 |  |  |  |  |
| Age |  |  |  |  | 0.025 | 0.101 | 3.054 | * |  |  |  |
| Education |  |  |  |  | 0.035 | 0.083 | 2.508 | * |  |  |  |
|  | *R^2^* | adj *R^2^* | *F*(3, 1007) | | *R^2^* | adj *R^2^* | *F*(5, 1005) | | *R^2^* change | *F* change | |
|  | 0.036 | 0.033 | 12.660 | ** | 0.048 | 0.043 | 10.071 | ** | 0.011 | 5.999 | * |
| **Personality factors** (Block 3; one by one) | | | | | | | | | | | |
| *Power to live* | β | std β | *ｔ* | | *R^2^* | adj *R^2^* | *F*(6, 938) | | *R^2^* change | *F* change | |
| Emotional regulation | 0.158 | 0.072 | 2.211 | * | 0.051 | 0.045 | 8.369 | ** | 0.005 | 4.891 | * |

Details are the same as for S5 Table.

**S7 Table.** **Facilitative factors for resolving general life difficulties by requesting help.**

| **Background factors** | | | | | | | | | | | |
| --- | --- | --- | --- | --- | --- | --- | --- | --- | --- | --- | --- |
|  | Block 1 (damage) | | | | + Block 2 (demographic) | | | | | | |
|  | β | std β | *ｔ* | | β | std β | *ｔ* | |  |  |  |
| Death of friend | 0.014 | 0.082 | 2.646 | * |  |  |  |  |  |  |  |
|  | *R^2^* | adj *R^2^* | *F*(1, 1026) | |  |  |  |  |  |  |  |
|  | 0.007 | 0.006 | 6.999 | * |  |  |  |  |  |  |  |
| **Personality factors** (Block 3; one by one) | | | | | | | | | | | |
| *Power to live* | β | std β | *ｔ* | | *R^2^* | adj *R^2^* | *F*(2, 948) | | *R^2^* change | *F* change | |
| Leadership | 0.030 | 0.068 | 2.067 | * | 0.011 | 0.009 | 5.459 | * | 0.004 | 4.271 | * |
| Altruism | 0.054 | 0.099 | 3.042 | ** | 0.017 | 0.014 | 7.968 | ** | 0.010 | 9.252 | ** |
| Stubbornness | 0.048 | 0.098 | 3.037 | ** | 0.017 | 0.014 | 7.952 | ** | 0.010 | 9.222 | ** |
| Emotional regulation | 0.035 | 0.068 | 2.082 | * | 0.011 | 0.009 | 5.492 | * | 0.005 | 4.335 | * |
| *Big 5* | β | std β | *ｔ* | | *R^2^* | adj *R^2^* | *F*(2, 959) | | *R^2^* change | *F* change | |
| Extraversion | 0.028 | 0.069 | 2.119 | * | 0.011 | 0.009 | 5.494 | * | 0.005 | 4.490 | * |
| Openness | 0.026 | 0.064 | 1.992 | * | 0.011 | 0.009 | 5.232 | * | 0.004 | 3.969 | * |

Details are the same as for S5 Table.

**S8 Table.** **Facilitative factors for resolving medico-psychological difficulties by requesting help.**

| **Background factors** | | | | | | | | | | | |
| --- | --- | --- | --- | --- | --- | --- | --- | --- | --- | --- | --- |
|  | Block 1 (damage) | | | | + Block 2 (demographic) | | | | | | |
|  | β | std β | *ｔ* | | β | std β | *ｔ* | |  |  |  |
| Difficulty experienced | 0.002 | 0.055 | 1.791 |  | 0.002 | 0.062 | 2.023 | * |  |  |  |
| Company housing  /official residence |  |  |  |  | 0.057 | 0.075 | 2.463 | * |  |  |  |
|  | *R^2^* | adj *R^2^* | F(1, 1073) | | *R^2^* | adj *R^2^* | *F*(2, 1072) | | *R^2^* change | *F* change | |
|  | 0.003 | 0.002 | 3.208 |  | 0.009 | 0.007 | 4.644 | * | 0.006 | 6.066 | * |
| **Personality factors** (Block 3; one by one) | | | | | | | | | | | |
| *Power to live* | β | std β | *ｔ* | | *R^2^* | adj *R^2^* | *F*(3, 993) | | *R^2^* change | *F* change | |
| Emotional regulation | 0.036 | 0.068 | 2.144 | * | 0.009 | 0.006 | 4.355 | * | 0.005 | 4.598 | * |
| Active well-being | 0.027 | 0.065 | 2.060 | * | 0.013 | 0.010 | 4.236 | * | 0.004 | 4.245 | * |

Details are the same as for S5 Table.

**S9 Table.** **Facilitative factors for resolving** **general life difficulties through family.**

| **Background factors** | | | | | | | | | | | |
| --- | --- | --- | --- | --- | --- | --- | --- | --- | --- | --- | --- |
|  | Block 1 (damage) | | | | + Block 2 (demographic) | | | | | | |
|  | β | std β | *ｔ* | | β | std β | *ｔ* | |  |  |  |
| Sex |  |  |  |  | -0.094 | -0.139 | -4.624 | ** |  |  |  |
| Age |  |  |  |  | -0.044 | -0.196 | -6.198 | ** |  |  |  |
| Three-generation |  |  |  |  | 0.081 | 0.100 | 3.234 | ** |  |  |  |
| Household others |  |  |  |  | -0.184 | -0.073 | -2.420 | * |  |  |  |
| Own house |  |  |  |  | 0.107 | 0.129 | 3.914 | ** |  |  |  |
| Company housing  /official residence |  |  |  |  | -0.216 | -0.070 | -2.281 | * |  |  |  |
|  |  |  |  |  | *R^2^* | adj *R^2^* | *F*(6, 1007) | |  |  |  |
|  |  |  |  |  | 0.095 | 0.089 | 17.546 | ** |  |  |  |
| **Personality factors** (Block 3; one by one) | | | | | | | | | | | |
| *Power to live* | β | std β | *ｔ* | | *R^2^* | adj *R^2^* | *F*(7, 929) | | *R^2^* change | *F* change | |
| Etiquette | -0.179 | -0.081 | -2.444 | * | 0.098 | 0.091 | 14.414 | ** | 0.006 | 5.972 | * |

Details are the same as for S5 Table.

**S10 Table.** **Facilitative factors for resolving** **medico-psychological difficulties through family.**

| **Background factors** | | | | | | | | | | | |
| --- | --- | --- | --- | --- | --- | --- | --- | --- | --- | --- | --- |
|  | Block 1 (damage) | | | | + Block 2 (demographic) | | | | | | |
|  | β | std β | *ｔ* | | β | std β | *ｔ* | |  |  |  |
| Household goods | 0.018 | 0.054 | 1.739 |  | 0.018 | 0.056 | 1.848 |  |  |  |  |
| Sex |  |  |  |  | -0.059 | -0.085 | -2.789 | * |  |  |  |
| Age |  |  |  |  | -0.026 | -0.113 | -3.719 | ** |  |  |  |
| Three-generation |  |  |  |  | 0.101 | 0.123 | 4.049 | ** |  |  |  |
|  | *R^2^* | adj *R^2^* | *F*(1, 1049) | | *R^2^* | adj *R^2^* | *F*(4, 1046) | | *R^2^* change | *F* change | |
|  | 0.003 | 0.002 | 3.025 |  | 0.041 | 0.038 | 11.304 | ** | 0.039 | 14.027 | ** |
| **Personality factors** (Block 3; one by one) | | | | | | | | | | | |
| *Power to live* | β | std β | *ｔ* | | *R^2^* | adj *R^2^* | *F*(5, 972) | | *R^2^* change | *F* change | |
| Problem solving | -0.138 | -0.062 | -1.980 | * | 0.040 | 0.035 | 8.180 | ** | 0.004 | 3.922 | * |
| Etiquette | -0.167 | -0.076 | -2.267 | * | 0.042 | 0.037 | 8.432 | ** | 0.005 | 5.138 | * |

Details are the same as for S5 Table.

**S11 Table.** **Facilitative factors for resolving** **general life difficulties through the help of an acquaintance.**

| **Background factors** | | | | | | | | | | | |
| --- | --- | --- | --- | --- | --- | --- | --- | --- | --- | --- | --- |
|  | Block 1 (damage) | | | | + Block 2 (demographic) | | | | | | |
|  | β | std β | *ｔ* | | β | std β | *ｔ* | |  |  |  |
| Refugee life | 0.041 | 0.082 | 2.560 | * | 0.046 | 0.092 | 2.902 | * |  |  |  |
| Age |  |  |  |  | -0.016 | -0.101 | -3.045 | * |  |  |  |
| Education |  |  |  |  | 0.030 | 0.109 | 3.263 | ** |  |  |  |
| Company housing  /official residence |  |  |  |  | 0.160 | 0.076 | 2.393 | * |  |  |  |
| Three-generation |  |  |  |  | 0.032 | 0.055 | 1.745 |  |  |  |  |
|  | *R^2^* | adj *R^2^* | *F*(1, 969) | | *R^2^* | adj *R^2^* | *F*(5, 965) | | *R^2^* change | *F* change | |
|  | 0.007 | 0.006 | 6.555 | * | 0.049 | 0.044 | 9.871 | ** | 0.042 | 10.635 | ** |
| **Personality factors** (Block 3; one by one) | | | | | | | | | | | |
| *Power to live* | β | std β | *ｔ* | | *R^2^* | adj *R^2^* | *F*(6, 894) | | *R^2^* change | *F* change | |
| Leadership | 0.159 | 0.126 | 3.829 | ** | 0.059 | 0.052 | 9.275 | ** | 0.015 | 14.664 | ** |
| Altruism | 0.142 | 0.093 | 2.837 | * | 0.052 | 0.045 | 8.123 | ** | 0.009 | 8.047 | * |
| Self-transcendence | 0.133 | 0.087 | 2.645 | * | 0.051 | 0.044 | 7.940 | ** | 0.007 | 6.998 | * |

Details are the same as for S5 Table.

**S12 Table.** **Facilitative factors for resolving** **medico-psychological difficulties through the help of an acquaintance.**

| **Background factors** | | | | | | | | | | | |
| --- | --- | --- | --- | --- | --- | --- | --- | --- | --- | --- | --- |
|  | Block 1 (damage) | | | | + Block 2 (demographic) | | | | | | |
|  | β | std β | *ｔ* | | β | std β | *ｔ* | |  |  |  |
| Household goods | 0.020 | 0.098 | 2.687 | * | 0.023 | 0.113 | 3.098 | * |  |  |  |
| Refugee life | 0.041 | 0.088 | 2.408 | * | 0.039 | 0.084 | 2.313 | * |  |  |  |
| Age |  |  |  |  | -0.018 | -0.124 | -3.930 | ** |  |  |  |
| Household income |  |  |  |  | 0.010 | 0.073 | 2.333 | * |  |  |  |
|  | *R^2^* | adj *R^2^* | *F*(2, 978) | | *R^2^* | adj *R^2^* | *F*(4, 976) | | *R^2^* change | *F* change | |
|  | 0.026 | 0.024 | 13.192 | ** | 0.048 | 0.044 | 12.282 | ** | 0.022 | 11.100 | ** |
| **Personality factors** (Block 3; one by one) | | | | | | | | | | | |
| *Power to live* | β | std β | *ｔ* | | *R^2^* | adj *R^2^* | *F*(5, 911) | | *R^2^* change | *F* change | |
| Leadership | 0.156 | 0.137 | 4.218 | ** | 0.063 | 0.058 | 12.304 | ** | 0.018 | 17.790 | ** |
| Altruism | 0.108 | 0.076 | 2.345 | * | 0.051 | 0.045 | 9.729 | ** | 0.006 | 5.497 | * |
| Emotional regulation | 0.094 | 0.071 | 2.183 | * | 0.050 | 0.045 | 9.576 | ** | 0.005 | 4.764 | * |
| Self-transcendence | 0.102 | 0.074 | 2.274 | * | 0.050 | 0.045 | 9.662 | ** | 0.005 | 5.172 | * |
| Active well-being | 0.084 | 0.082 | 2.544 | * | 0.052 | 0.046 | 9.934 | ** | 0.007 | 6.473 | * |
| *Big 5* | β | std β | *ｔ* | | *R^2^* | adj *R^2^* | *F*(5, 913) | | *R^2^* change | *F* change | |
| Extraversion | 0.142 | 0.134 | 4.157 | ** | 0.061 | 0.056 | 11.918 | ** | 0.018 | 17.283 | ** |
| Neuroticism | -0.105 | -0.092 | -2.813 | * | 0.052 | 0.047 | 9.959 | ** | 0.008 | 7.916 | * |
| Openness | 0.071 | 0.068 | 2.099 | * | 0.048 | 0.043 | 9.224 | ** | 0.005 | 4.404 | * |

Details are the same as for S5 Table.

**S13 Table.** **Facilitative factors for resolving** **general life difficulties through cooperation.**

| **Background factors** | | | | | | | | | | | |
| --- | --- | --- | --- | --- | --- | --- | --- | --- | --- | --- | --- |
|  | Block 1 (damage) | | | | + Block 2 (demographic) | | | | | | |
|  | β | std β | *ｔ* | | β | std β | *ｔ* | |  |  |  |
| Household goods | 0.019 | 0.093 | 2.635 | * |  |  |  |  |  |  |  |
| Refugee life | 0.082 | 0.181 | 5.230 | ** |  |  |  |  |  |  |  |
| Difficulty experienced | 0.012 | 0.069 | 2.203 | * |  |  |  |  |  |  |  |
|  | *R^2^* | adj *R^2^* | *F*(3, 1004) | |  |  |  |  |  |  |  |
|  | 0.068 | 0.065 | 24.299 | ** |  |  |  |  |  |  |  |
| **Personality factors** (Block 3; one by one) | | | | | | | | | | | |
| *Power to live* | β | std β | *ｔ* | | *R^2^* | adj *R^2^* | *F*(4, 931) | | *R^2^* change | *F* change | |
| Emotional regulation | 0.113 | 0.084 | 2.681 | * | 0.079 | 0.075 | 19.867 | ** | 0.007 | 7.188 | * |
| Active well-being | 0.080 | 0.077 | 2.443 | * | 0.077 | 0.073 | 19.538 | ** | 0.006 | 5.969 | * |

Details are the same as for S5 Table.

**S14 Table.** **Facilitative factors for resolving medico-psychological difficulties through cooperation.**

| **Background factors** | | | | | | | | | | | |
| --- | --- | --- | --- | --- | --- | --- | --- | --- | --- | --- | --- |
|  | Block 1 (damage) | | | | + Block 2 (demographic) | | | | | | |
|  | β | std β | *ｔ* | | β | std β | *ｔ* | |  |  |  |
| Car | 0.030 | 0.077 | 2.507 | * |  |  |  |  |  |  |  |
| Refugee life | 0.058 | 0.140 | 4.537 | ** |  |  |  |  |  |  |  |
| Difficulty experienced | 0.009 | 0.122 | 3.905 | ** |  |  |  |  |  |  |  |
|  | *R^2^* | adj *R^2^* | *F*(3, 1044) | |  |  |  |  |  |  |  |
|  | 0.053 | 0.050 | 19.546 | ** |  |  |  |  |  |  |  |
| **Personality factors** (Block 3; one by one) | | | | | | | | | | | |
| *Power to live* | β | std β | *ｔ* | | *R^2^* | adj *R^2^* | *F*(4, 970) | | *R^2^* change | *F* change | |
| Emotional regulation | 0.097 | 0.082 | 2.627 | * | 0.064 | 0.060 | 16.472 | ** | 0.007 | 6.900 | * |
| Active well-being | 0.072 | 0.078 | 2.504 | * | 0.063 | 0.059 | 16.306 | ** | 0.006 | 6.272 | * |
| *Big 5* | β | std β | *ｔ* | | *R^2^* | adj *R^2^* | *F*(4, 972) | | *R^2^* change | *F* change | |
| Neuroticism | -0.083 | -0.084 | -2.680 | * | 0.058 | 0.055 | 15.085 | ** | 0.007 | 7.180 | * |

Details are the same as for S5 Table.

**S15 Table.** **Facilitative factors for resolving** **general life difficulties through public assistance.**

| **Background factors** | | | | | | | | | | | |
| --- | --- | --- | --- | --- | --- | --- | --- | --- | --- | --- | --- |
|  | Block 1 (damage) | | | | + Block 2 (demographic) | | | | | | |
|  | β | std β | *ｔ* | | β | std β | *ｔ* | |  |  |  |
| Household goods | 0.039 | 0.158 | 4.445 | ** | 0.041 | 0.166 | 4.670 | ** |  |  |  |
| Death of a family member | 0.067 | 0.076 | 2.452 | * | 0.069 | 0.078 | 2.527 | * |  |  |  |
| Refugee life | 0.080 | 0.148 | 4.215 | ** | 0.076 | 0.141 | 4.040 | ** |  |  |  |
| Difficulty experienced | 0.012 | 0.060 | 1.908 |  | 0.014 | 0.070 | 2.236 | * |  |  |  |
| Age |  |  |  |  | -0.012 | -0.069 | -2.099 | * |  |  |  |
| Student |  |  |  |  | 0.101 | 0.066 | 2.025 | * |  |  |  |
|  | *R^2^* | adj *R^2^* | *F*(4, 959) | | *R^2^* | adj *R^2^* | *F*(6, 957) | | *R^2^* change | *F* change | |
|  | 0.090 | 0.086 | 23.678 | ** | 0.102 | 0.096 | 18.108 | ** | 0.012 | 6.433 | * |
| **Personality factors** (Block 3; one by one) | | | | | | | | | | | |
| *Power to live* | β | std β | *ｔ* | | *R^2^* | adj *R^2^* | *F*(7, 888) | | *R^2^* change | *F* change | |
| Emotional regulation | 0.145 | 0.094 | 2.944 | ** | 0.118 | 0.111 | 16.957 | ** | 0.009 | 8.669 | ** |
| *Big 5* | β | std β | *ｔ* | | *R^2^* | adj *R^2^* | *F*(7, 894) | | *R^2^* change | *F* change | |
| Neuroticism | -0.128 | -0.094 | -2.946 | ** | 0.111 | 0.104 | 15.916 | ** | 0.009 | 8.679 | ** |

Details are the same as for S5 Table.

**S16 Table.** **Facilitative factors for resolving** **medico-psychological difficulties through public assistance.**

| **Background factors** | | | | | | | | | | | |
| --- | --- | --- | --- | --- | --- | --- | --- | --- | --- | --- | --- |
|  | Block 1 (damage) | | | | + Block 2 (demographic) | | | | | | |
|  | β | std β | *ｔ* | | β | std β | *ｔ* | |  |  |  |
| Household goods | 0.020 | 0.087 | 2.481 | * | 0.021 | 0.089 | 2.536 | * |  |  |  |
| Refugee life | 0.059 | 0.115 | 3.260 | ** | 0.058 | 0.112 | 3.176 | ** |  |  |  |
| Couple |  |  |  |  | -0.038 | -0.063 | -2.065 | * |  |  |  |
|  | *R^2^* | adj *R^2^* | *F*(2, 1040) | | *R^2^* | adj *R^2^* | *F*(3, 1039) | | *R^2^* change | *F* change | |
|  | 0.031 | 0.029 | 16.578 | ** | 0.035 | 0.032 | 12.509 | ** | 0.004 | 4.266 | * |
| **Personality factors** (Block 3; one by one) | | | | | | | | | | | |
| *Power to live* | β | std β | *ｔ* | | *R^2^* | adj *R^2^* | F(4, 965) | | *R^2^* change | *F* change | |
| Emotional regulation | 0.125 | 0.086 | 2.722 | * | 0.042 | 0.038 | 10.537 | ** | 0.007 | 7.411 | * |
| Self-transcendence | 0.097 | 0.063 | 2.004 | * | 0.038 | 0.035 | 9.658 | ** | 0.004 | 4.017 | * |
| *Big 5* | β | std β | *ｔ* | | *R^2^* | adj *R^2^* | F(4, 968) | | *R^2^* change | *F* change | |
| Agreeableness | 0.087 | 0.063 | 2.020 | * | 0.046 | 0.042 | 11.639 | ** | 0.004 | 4.082 | * |
| Neuroticism | -0.085 | -0.068 | -2.173 | * | 0.047 | 0.043 | 11.806 | ** | 0.005 | 4.721 | * |

Details are the same as for S5 Table.
