# Supplementary material for: Self-help and mutual assistance in the aftermath of a tsunami: How individual factors contribute to resolving difficulties
Source: PLoS One. 2021 Oct 7;16(10):e0258325. doi: 10.1371/journal.pone.0258325 (PMC8496872; doi:10.1371/journal.pone.0258325)
Supplement: S1 File — (PDF) [file pone.0258325.s003.pdf]

考え方・習慣と東日本大震災での経験についてのアンケート

このアンケートは、ご自身の性格や考え方、日ごろの習慣などについてお答え頂くとともに、東日本大震災の様々な、大変なご経験についておたずねします。

アンケートの質問は、東日本大震災で被災し、様々な苦労をされた方々を対象として平成24年度に実施したインタビュー調査をもとに作成したものです。

人間の性格や考え方、日ごろの習慣について、震災での様々な経験の中で複数の方が「重要だと思う」あるいは「よくない」と述べられたことなどをまとめました。そういったご意見について、みなさまがどうお考えになるかを調べたいと思っています。

●記入にあたってのお願い●

1．大変申し訳ありませんが、質問数が多くなっていますので、途中で休憩を入れながらご自身のペースで回答をしてください。

2．質問文を読み、あてはまる選択肢の番号を○で囲んでください。

3．ご記憶がややあいまいな場合や、あてはまる回答に迷われる場合は、およその目安や、お考えに近いものをご回答ください。あまり深く考え込まずに、感じるまま・思うままに素直に回答してください。

4．○は１つだけ、いくつでも、など回答数の指示があります。よく読んでお答えください。

5．（　　）内には、該当する数字や言葉を具体的にお書きください。

6．筆記用具（鉛筆・ボールペン等）は特に指定はありません。ご回答が読み取りやすいように、はっきりとご記入ください。

＝　回答の記入例　＝

問０　あなたの性格についておたずねします。
各項目について、右の０～５の数字のうち、あてはまるもの１つに○をつけてください。

	全くあてはまらない					非常にあてはまる
（1）　買い物で商品を選ぶときに、長い時間迷ってしまう	(0)	1	2	3	4	5
（2）　予定時刻に遅れないように、時間の余裕を考えて行動する	0	1	2	3	(4)	5

問０　あなたの性別をお答えください。

1．男　性　　　　(2) 女　性

問０　それはいつ頃のことでしょうか。

震災からおよそ（　1　）年（　8　）か月後

まず最初に、ご自身の性格についておたずねします。

問１　あなたの性格についておたずねします。
各項目について、右の０～５の数字のうち、あてはまるもの１つに○をつけてください。

	全くあてはまらない					非常にあてはまる
（1）　活発で、外向的だと思う	0	1	2	3	4	5
（2）　ひかえめで、おとなしいと思う	0	1	2	3	4	5
（3）　人に気をつかう、やさしい人間だと思う	0	1	2	3	4	5
（4）　他人に不満をもち、もめごとを起こしやすいと思う	0	1	2	3	4	5
（5）　しっかりしていて、自分に厳しいと思う	0	1	2	3	4	5
（6）　だらしなく、うっかりしていると思う	0	1	2	3	4	5
（7）　心配性で、うろたえやすいと思う	0	1	2	3	4	5
（8）　冷静で、気分が安定していると思う	0	1	2	3	4	5
（9）　新しいことが好きで、変わった考えをもつと思う	0	1	2	3	4	5
（10）　発想力に欠けた、平凡な人間だと思う	0	1	2	3	4	5
（11）　困っている人を見ると放っておけない	0	1	2	3	4	5
（12）　おせっかいで、人に世話を焼くのが好きである	0	1	2	3	4	5
（13）　さみしがり屋で、一人でいるのは好きでない	0	1	2	3	4	5
（14）　他人の幸福が嬉しいので、人助けをする	0	1	2	3	4	5
（15）　人から頼られたり感謝されるのが好きである	0	1	2	3	4	5
（16）　人から頼まれると、どうしても断れない	0	1	2	3	4	5
（17）　負けず嫌いである	0	1	2	3	4	5
（18）　頑固で、自分の意思を通す	0	1	2	3	4	5
（19）　自分の好きなもの、やりたいことに対する欲が強い	0	1	2	3	4	5
（20）　自分はなんとかなる、という自信がある	0	1	2	3	4	5
（21）　言いたいことはその場で言ってしまう	0	1	2	3	4	5
（22）　だめなものはだめ、いいものはいい、と白黒はっきりさせる	0	1	2	3	4	5
（23）　周りの人が慌てているときほど、なぜか冷静になる	0	1	2	3	4	5

次に、ご自身の考え方や習慣についておたずねします。

問２　以下に述べられている意見は、あなたの日頃の考え方や習慣にどれくらい当てはまりますか。
各項目について、右の１～７の数字のうち、あてはまるもの１つに〇をつけてください。

	全くあてはまらない					非常にあてはまる
（1）　人によって考え方が違うのは仕方がないと思う	0	1	2	3	4	5
（2）　社会の中で自分が果たすべき役割を認識している	0	1	2	3	4	5
（3）　他者への行いは、巡り巡って自分に戻ってくると思う	0	1	2	3	4	5
（4）　自分が生きている、生かされている、ことを意識している	0	1	2	3	4	5
（5）　人として従うべき道や教えを認識している	0	1	2	3	4	5
（6）　何かあったときに、慌てず冷静でいられるよう心掛ける	0	1	2	3	4	5
（7）　辛い時に、くよくよ考えないように努力する	0	1	2	3	4	5
（8）　辛い時に、周りや社会の状況と自分を比較して、仕方がないと考える	0	1	2	3	4	5
（9）　辛い時に、これが将来自分のプラスになると思って前向きに取り組む	0	1	2	3	4	5
（10）　問題を解決するために、まず自分から動く	0	1	2	3	4	5
（11）　何をすべきか悩むとき、いくつかの選択肢を比較する	0	1	2	3	4	5
（12）　行動に移る前に、優先順位や段取りを考える	0	1	2	3	4	5
（13）　自分から人に話し掛ける	0	1	2	3	4	5
（14）　困ったときは、すぐ人にお願いする	0	1	2	3	4	5
（15）　人にお世話になったときは、はっきりと感謝の気持ちを伝える	0	1	2	3	4	5
（16）　弱い気持ちを顔に出さないように努力する	0	1	2	3	4	5
（17）　人の心を動かす、気のきいた言葉が口から出てくる	0	1	2	3	4	5
（18）　問題解決のためには、自分から関係者を集めて話し合いをする	0	1	2	3	4	5
（19）　自分の立場や、周囲に与える印象を悪くしないように気を配る	0	1	2	3	4	5

	全くあてはまらない					非常にあてはまる
（20） 話をするとき、相手の性格・願望・能力を考え、適切な言葉や態度を選ぶ	0	1	2	3	4	5
（21） 家庭環境や日常生活の経験で、よい性格や考え方が身に付いた	0	1	2	3	4	5
（22） 両親・家族や近所の人からのしつけ・教育によって、よい性格や考え方が身に付いた	0	1	2	3	4	5
（23） 学校・塾などの授業や教師の指導で、よい性格や考え方が身に付いた	0	1	2	3	4	5
（24） 運動・スポーツ・武道等の経験を通じて、よい性格や考え方が身に付いた	0	1	2	3	4	5
（25） 読書や人の話によって、よい性格や考え方が身に付いた	0	1	2	3	4	5
（26） 仕事の経験を通じて、よい性格や考え方が身に付いた	0	1	2	3	4	5
（27） 日頃、気分転換やストレス解消のための習慣を欠かさない	0	1	2	3	4	5
（28） 日頃、新しい知識・技術・考え方を身に付ける機会を持つようにしている	0	1	2	3	4	5
（29） 日頃、身体の健康を維持するための習慣を欠かさない	0	1	2	3	4	5
（30） 日頃、なるべく自分のことは自分でしている	0	1	2	3	4	5
（31） 日常、家族や近所の人に自分から挨拶をしている	0	1	2	3	4	5
（32） 日頃、友人・知人との連絡を欠かさないようにしている	0	1	2	3	4	5
（33） 日頃、自分から声をかけて集団をまとめることが多い	0	1	2	3	4	5

2011 年 3 月 11 日の地震発生【以前】のことについておたずねします。

問３−１　2011年3月11日の地震発生以前、身体の状態はいかがでしたか。(〇は当てはまる程度の数字にひとつ)

健康だった　　　　　　　　　　　　　　非常に悪かった

0 ──── 1 ──── 2 ──── 3 ──── 4 ──── 5

問３−２　地震発生以前、心の状態はいかがでしたか。(〇はひとつ)

健康だった　　　　　　　　　　　　　　非常に悪かった

0 ──── 1 ──── 2 ──── 3 ──── 4 ──── 5

問３−３　地震発生以前、運動の習慣はありましたか。(〇はひとつ)

1. ほぼ毎日　2. ときどき　3. ほとんどなかった　4. 全くなかった

問３−４　地震発生以前、飲酒の習慣はありましたか。(〇はひとつ)

1. ほぼ毎日　2. ときどき　3. ほとんどなかった　4. 全くなかった

問３−５　地震発生以前、経済的な不安はありましたか。(〇はひとつ)

なかった　　　　　　　　　　　　　　非常に強く感じていた

0 ──── 1 ──── 2 ──── 3 ──── 4 ──── 5

問３−６　地震発生以前、家族との関係はどうでしたか。(〇はひとつ)

1. 良かった　2. やや良かった　3. やや悪かった
4. 悪かった　5. 家族はいなかった

問３−７　地震発生以前、親族との関係はどうでしたか。(〇はひとつ)

1. 良かった　2. やや良かった　3. やや悪かった
4. 悪かった　5. 親族はいなかった

問３−８　地震発生以前、友人との関係はどうでしたか。(〇はひとつ)

1. 良かった　2. やや良かった　3. やや悪かった
4. 悪かった　5. 友人はいなかった

問３−９　地震発生以前、職場の人間関係はどうでしたか。(〇はひとつ)

1. 良かった　2. やや良かった　3. やや悪かった
4. 悪かった　5. 仕事はしていなかった

問３-10　地震発生以前、近所にいる友人・知人はどのくらいでしたか。（○はひとつ）

1. とても多かった	2. やや多かった	3. 少なかった	4. いなかった

問３-11　地震発生以前、世帯での自家用車の保有台数は何台でしたか。（○はひとつ）

1. ０台	2. １台	3. ２台以上

問３-12　地震発生以前、地域の避難・防災訓練はどのくらいの頻度で開催されていましたか。（○はひとつ）

1. 開催されていなかった	2. 年に１回程度	3. 年に2回程度	4. 年に3回以上

問３-13　地震発生以前、あなたご自身の避難・防災訓練への参加頻度はどのくらいでしたか。（○はひとつ）

1. 参加したことがなかった	2. 年に１回程度	3. 年に2回程度	4. 年に3回以上

問３-14　地震発生以前、キャンプ・野外炊飯等の経験はありましたか。（○はひとつ）

1. たくさんあった	2. 少しあった	3. ない

問３-15　地震発生以前、住んでいる地域に過去の震災を伝える記念碑などの歴史物があることを知っていましたか。（○はひとつ）

1. 知っていた	2. 知らなかった	3. 歴史物はない

問３-16　地震発生以前、親族から過去の津波の経験談を聞いたことがありましたか。（○はひとつ）

1. よく聞いていた	2. 聞いたことがあった	3. 全く聞いたことがなかった

問３-17　2011年3月11日の津波以前、過去の津波で次のような経験をしたことがありますか。（あてはまるものすべてに○）

1. 津波に巻き込まれたことがあった	2. 自宅が浸水したことがあった
3. 実際に津波を見たことがあった	4. いずれもなかった

問３-18　2011年3月11日の津波以前、過去の津波で次のような避難の体験はありますか。（○はひとつ）

1. 避難して津波から逃れられた	2. 津波が来たが避難しなかった
3. 避難したが津波は来なかった	4. 避難する機会がなかった

問３-19　2011年3月11日の津波以前、津波の発生の仕組みを知っていましたか。（○はひとつ）

1. 説明できる程度詳しく知っていた	2. 知っていた
3. あまり知らなかった	4. 全く知らなかった

2011 年 3 月 11 日の地震と津波による直接の被害についておたずねします。

問４－１　自宅の建物の被害は、どの程度でしたか。（○はひとつ）

1. 被害なし
2. 床下浸水
3. 床上浸水
4. 一部損壊
5. 半壊
6. 大規模半壊
7. 全壊(津波で流失した場合も含む)

問４－２　自宅の家財の被害は、どの程度でしたか。（○はひとつ）

1. 被害なし
2. 一部被害
3. 半分程度被害
4. ほぼすべて被害

問４－３　世帯で所有していた車の被害は、どの程度でしたか。（○はひとつ）

1. 使える車を全て失った
2. 使える車があった
3. 車を所有していなかった

問４－４　自分の負傷は、どの程度でしたか。（○はひとつ）

1. なし
2. 負傷したが通院する程度ではなかった
3. 通院するほどの負傷だった
4. 入院するほどの負傷だった

問４－５　家族の中で次のような方はいらっしゃいますか。（あてはまるものすべてに○）

1. 通院する程度ではない負傷をされた方
2. 通院するほどの負傷をされた方
3. 入院するほどの負傷をされた方
4. 震災が原因で亡くなられた方
5. （1～4にあてはまる人は）いない

問４－６　仲の良い友人の中で次のような方はいらっしゃいますか。（あてはまるものすべてに○）

1. 通院する程度ではない負傷をされた方
2. 通院するほどの負傷をされた方
3. 入院するほどの負傷をされた方
4. 震災が原因で亡くなられた方
5. （1～4にあてはまる人は）いない

問４－７　震災前の職業にどのような影響がありましたか。（あてはまるものすべてに○）

1. 震災による影響は全くない
2. 一時的に仕事を中断した
3. しばらく仕事を中断した
4. 職を失うこととなった
5. 震災前、職業についていなかった

2011 年 3 月 11 日の地震後の津波避難についておたずねします。

問５－１　2011年3月11日の地震発生の際、あなたは津波に対する避難をしましたか。

1. した	2. しなかった

⇩ 問 5-2 以降にお答えください

↳ 10 ページの問 6-1 に進んでください

問５－２　地震直後の様子や行動などについておたずねします。
各項目について、あてはまるもの１つに〇をつけてください。

		はい	いいえ
（1）	揺れを感じたとき、そこに津波が来る危険があると思いましたか	1	2
（2）	揺れの直後、津波が来る恐怖を感じましたか	1	2
（3）	揺れの直後、過去の津波のことを思い出しましたか<br>（※過去に津波の経験が「ない」場合、２を選んでください）	1	2
（4）	揺れの直後、避難訓練・防災訓練のことを思い出しましたか<br>（※過去に避難訓練・防災訓練の経験が「ない」場合、２を選んでください）	1	2
（5）	揺れの直後、すぐに避難しましたか	1	2
（6）	揺れの直後、誰かに促される前に自主的に避難しましたか	1	2
（7）	揺れの直後、家族や知人に避難を促されましたか	1	2
（8）	揺れの直後、誰かの助言が、避難行動の助けになりましたか	1	2
（9）	揺れの直後、家族と連絡をとろうとしましたか	1	2
（10）	揺れの直後、すぐに家族の安否が確認できましたか	1	2
（11）	揺れの直後、倒れたものや壊れたものの後片づけをしましたか	1	2

問５－３　揺れの直後、次のような手段で避難を呼びかける情報（避難勧告）を聞きましたか。

（あてはまるものすべてに〇）

1. ラジオ	2. テレビ
3. 携帯電話	4. 防災行政無線
5. その他（　　　　　　　　　　）	6. 聞かなかった

問５−４　揺れの直後、周囲の道路に次のような被害を見ましたか。（あてはまるものにすべてに○）

1. 液状化
2. 小さい亀裂
3. 大きい亀裂
4. 陥没
5. 見なかった

問５−５　避難の際の様子や行動などについておたずねします。
各項目について、あてはまるもの１つに○をつけてください。

	はい	いいえ
（1）　避難行動を行ったのは津波を見てからですか	1	2
（2）　自動車で避難しましたか	1	2
（3）　避難する途中に、他者から声掛けをされましたか	1	2
（4）　避難する途中に、他者に助けられましたか	1	2
（5）　避難する途中に、他の人にも避難するように声を掛けましたか	1	2
（6）　避難する途中に、他の人を助けましたか	1	2
（7）　避難する途中に、絶対に生き延びるという強い気持ちがありましたか	1	2
（8）　避難する途中に、頼りになる人がいましたか	1	2
（9）　避難する際、できるだけ高いところを目指しましたか	1	2

問５−６　振り返ってみると、あなたの避難行動は適切でしたか。（○はひとつ）

1. 適切であった
2. どちらかといえば適切であった
3. どちらともいえない
4. どちらかといえば不適切であった
5. 不適切であった

地震・津波（あるいは避難）の後から、通常の生活の再開あるいは仮設住宅生活に入るまでの間についておたずねします。

問６－１　以下のことがらについて、困ったことはありましたか。困った程度に○をつけ、「少し」あるいは「大変困った」と答えた項目については、解決の方法・経緯についても、あてはまる選択肢に○をつけてください。

	困った程度（○はひとつ）			困った場合、解決方法・経緯（あてはまるものすべてに○）						
	困らなかった	少し困った	大変困った	自分自身の工夫	自分から人に依頼	家族・親族の助け	知人の助け	避難者同士の協力	公共（自治体・自衛隊・ボランティア）の助け	解決しなかった
（1）　食べる	0	1	2	a	b	c	d	e	f	g
（2）　料理する	0	1	2	a	b	c	d	e	f	g
（3）　適当な着替え	0	1	2	a	b	c	d	e	f	g
（4）　室温	0	1	2	a	b	c	d	e	f	g
（5）　睡眠	0	1	2	a	b	c	d	e	f	g
（6）　トイレ	0	1	2	a	b	c	d	e	f	g
（7）　洗面	0	1	2	a	b	c	d	e	f	g
（8）　入浴	0	1	2	a	b	c	d	e	f	g
（9）　洗濯	0	1	2	a	b	c	d	e	f	g
（10）情報収集	0	1	2	a	b	c	d	e	f	g
（11）交通移動	0	1	2	a	b	c	d	e	f	g
（12）自身の医療・介護	0	1	2	a	b	c	d	e	f	g
（13）家族の医療・介護	0	1	2	a	b	c	d	e	f	g
（14）自身の精神の安定	0	1	2	a	b	c	d	e	f	g
（15）家族の精神の安定	0	1	2	a	b	c	d	e	f	g
（16）騒音	0	1	2	a	b	c	d	e	f	g
（17）悪臭	0	1	2	a	b	c	d	e	f	g
（18）プライバシー	0	1	2	a	b	c	d	e	f	g

**問６－２　以下のことがらについて困っている他の人を（直接的・間接的に）助ける機会がありましたか？
助ける機会があった場合は「1.あった」に○をつけ、その問題の解決の方法・経緯についても、あてはまる選択肢に○つけてください。助ける機会や余裕がなかった場合は、「0.なかった」に○をつけてください。**

	助ける機会（○はひとつ）		助ける機会があった場合 解決方法・経緯（あてはまるものすべてに○）	
	なかった	あった	自分自身の工夫で解決	自分から人に頼んで助けてもらった
（1）　食べる	0	1	a	b
（2）　料理する	0	1	a	b
（3）　適当な着替え	0	1	a	b
（4）　室温	0	1	a	b
（5）　睡眠	0	1	a	b
（6）　トイレ	0	1	a	b
（7）　洗面	0	1	a	b
（8）　入浴	0	1	a	b
（9）　洗濯	0	1	a	b
（10）　情報収集	0	1	a	b
（11）　交通移動	0	1	a	b
（12）　医療・介護	0	1	a	b
（13）　精神の安定	0	1	a	b
（14）　騒音	0	1	a	b
（15）　悪臭	0	1	a	b
（16）　プライバシー	0	1	a	b

問６－３　災害への備えと、その結果について当てはまる選択肢に○をつけてください。

	備えがあり役に立った	備えはあったが役に立たなかった・使えなかった	備えはあったが必要がなかった	備えがなく困った	備えはなかったが困らなかった
（1）　飲料水	1	2	3	4	5
（2）　食料	1	2	3	4	5
（3）　燃料	1	2	3	4	5
（4）　ラジオ	1	2	3	4	5
（5）　懐中電灯	1	2	3	4	5
（6）　携帯型発電機	1	2	3	4	5
（7）　燃料式発電機	1	2	3	4	5
（8）　携帯型トイレ	1	2	3	4	5

問６－４　避難後、少し落ち着いてから、「あってよかった」、「助かった」と思ったことは何ですか。

（あてはまるものにすべてに○）

1.　テレビなどの娯楽	2.　趣味などの生きがい
3.　仕事の継続	4.　ボランティアなどほかの地域の人の支援
5.　避難者同士の助け合い	6.　自衛隊の助け
7.　仮設住宅や避難所の快適性	8.　衛生的な環境
9.　お風呂・入浴	10.　化粧用品
11.　散髪	12.　プライバシー
13.　穏やかな人間関係	14.　避難者同士の会話
15.　警察や消防などの巡回	16.　公共交通機関（電車・バス等）
17.　自家用車	18.　大工さん・電気屋さん等のプロの技
19.　医療施設	20.　その他（　　　　　　　　　　）
21.　特にない	

現在の生活についておたずねします。

問７－１　震災前の生活を100%として、(1) 震災直後に生活は何%まで損なわれ、(2) 現在の生活は何%まで回復したと感じますか。0以上の数字でお答えください。

（※直後から全く生活が損なわれなかったとお考えになる方は、全て100%とお答えください）

（1）震災直後の生活　[　|　|　] ％

（2）現在の生活　[　|　|　] ％

問７－２　復興へ向けて、あなたの意欲はどのくらいですか。（○はひとつ）

1. とても高い　　2. 高い
3. どちらともいえない　　4. 低い
5. とても低い

問７－３　被災や避難生活のストレスを、現在も感じますか。（○はひとつ）

1. 強く感じている　　2. 感じている
3. どちらともいえない　　4. 感じていない
5. 全く感じていない

【責任者であった事業が被災により中断した方のみお答えください】
問７－４　事業はいつごろ再開できましたか。

震災からおよそ（　　　　　）年（　　　　　）か月後

【被災により仕事（雇用）を失った方のみお答えください】
問７－５　いつごろ新しい仕事（雇用）を得ましたか。

震災からおよそ（　　　　　）年（　　　　　）か月後

問７－６　現在の居住環境は快適ですか。（○はひとつ）

1. とても快適　　2. 快適
3. どちらともいえない　　4. 不快
5. とても不快

【ご自宅を失った方のみお答えください】
問７－７　ご自宅の再建状況はいかがですか。（○はひとつ）

1. 再建した　　2. 再建の予定あり　　3. 再建の予定なし

【問７－７で「1.再建した」、「2.再建の予定あり」と回答した方のみお答えください】
問７－８　いつごろ再建できましたか、あるいはできそうですか。

1. 震災からおよそ（　　　　　）年（　　　　　）か月後　　2. 時期は未定

【自宅の片づけ・一応の修復をする必要があった方のみお答えください】

問７－９　自宅の片づけ・一応の修復が完了するスピードはいかがでしたか。

1. 予想より時間がかからなかった	2. 予想した通りだった
3. 予想より時間がかかった	4. まだ終わっていない

【問７－９で「1」～「3」と回答した方のみお答えください】

問７-10　自宅の片づけ・一応の修復はいつごろ完了しましたか。

震災からおよそ（　　　　　）年（　　　　　）か月後

【自動車を流失した方のみお答えください】

問７-11　自動車の再入手状況はいかがですか。（○はひとつ）

1. 再入手した	2. 再入手の予定あり	3. 再入手の予定なし

【問７-11で「1.再入手した」、「2.再入手の予定あり」と回答した方のみお答えください】

問７-12　いつごろ入手できましたか、あるいはできそうですか。

1. 震災からおよそ（　　　　　）年（　　　　　）か月後	2. 時期は未定

問７-13　他の被災者を助ける活動に参加されてますか。（○はひとつ）

1. 積極的に参加している	2. 参加したことはある	3. 参加したことはない

問７-14　今回の被災経験を伝える活動に参加されていますか。（○はひとつ）

1. 積極的に参加している	2. 参加したことはある	3. 参加したことはない

問７-15　次の震災への備えをしていますか。（あてはまるものすべてに○）

1. 飲料水	2. 食料
3. 燃料	4. ラジオ
5. 懐中電灯	6. 携帯型発電機
7. 燃料式発電機	8. 携帯型トイレ
9. 高台移転	10. 耐震補強
11. 地震保険加入	12. その他（　　　　　　　　　　）
13. していない	

問７-16　現在、趣味や楽しみはありますか。（○はひとつ）

1. ある（震災発生前と同じ）	2. ある（震災発生前と異なる）
3. ない（震災発生前からない）	4. ない（震災がきっかけでできなくなったしまった）

問７−17　ここ１年間の、地域の活動などへのあなたの参加状況ついておたずねします。
各項目について、あてはまるもの１つに○をつけてください。

	よく参加している	ときどき参加している	参加していない	参加する機会がない
（1）　地域の祭り	1	2	3	4
（2）　防犯・防災活動	1	2	3	4
（3）　ボランティア活動	1	2	3	4
（4）　学校のＰＴＡ活動	1	2	3	4
（5）　子ども会活動	1	2	3	4
（6）　学校の行事（運動会・文化祭）	1	2	3	4
（7）　地域の講演会・勉強会	1	2	3	4
（8）　趣味・サークル活動	1	2	3	4
（9）　被災者支援の各種のイベント	1	2	3	4

問７−18　現在のお住まいの場所でのご近所様とのご関係はいかがですか。（○はひとつ）

1. 良い
2. どちらかといえば良い
3. どちらでもない
4. どちらかといえば悪い
5. 悪い

問７−19　その現在の近所のお住まいの方々は、震災前の知り合いと同じですか。（○はひとつ）

1. 全く同じ
2. ほぼ同じ
3. 一部同じ
4. 全く異なる

【震災前に就業されていた方のみお答えください】
問７−20　震災によるご職業への影響や現在の状況についてお聞かせください。（○はひとつ）

1. 全く同じ
2. ほぼ同じ
3. 一部同じ
4. 全く異なる

問７−21　この1年間（報酬の有無に関わらず）お仕事の忙しさはいかがですか。（○はひとつ）

1. とても忙しい
2. 忙しい
3. どちらかといえば忙しい
4. どちらかといえば忙しくはない
5. 忙しくない
6. 仕事はしていない

問７−22　震災発生から現在までのお住まいの自治体の対応はいかがですか。（○はひとつ）

1. とても満足である
2. 満足である
3. どちらかといえば満足である
4. どちらかといえば不満である
5. 不満である
6. とても不満である

問７-23　現在の交通の便はいかがですか。(○はひとつ)

1. とても便利である
2. 便利である
3. どちらかといえば便利である
4. どちらかといえば不便である
5. 不便である
6. とても不便である

問７-24　現在おすまいの地域（市または町全体）の復旧・復興の状況をどのように感じていますか。（○はひとつ）

1. とても速い
2. 速い
3. ふつう
4. 遅い
5. とても遅い
6. 震災の影響はない

問７-25　次のようなご友人・知人の方は，あなたの周りにいらっしゃいますか。(あてはまるものすべてに○)

1. 一緒にいると楽しい人
2. 信頼できる・頼りになる人
3. よくお話する相手
4. 励ましたり・応援してくれる人
5. 困ったときに助けてくれる人
6. 生活再建等に必要な情報を教えてくれる(口コミしてくれる)人

問７-26　震災後、今生きていることの「使命感」を感じることはありますか。(○はひとつ)

1. いつも感じている
2. しばしば感じている
3. 感じたことがある
4. 感じたことはない
5. まったく感じたことはない

【ご家族を亡くされた方のみお答えください】
問７-27　葬儀を執り行うことができましたか。(○はひとつ)

1. できた
2. できなかった

【問７-27で「1.できた」と回答した方のみお答えください】
問７-28　執り行った葬儀についてどう思っていますか。(○はひとつ)

1. かなり不満がある
2. やや不満がある
3. どちらでもない
4. あまり不満はない
5. まったく不満はない

問７-29　１週間のうち外出する日はどれぐらいですか。(○はひとつ)

1. 毎日
2. 週５～６日
3. 週３～４日
4. 週１～２日
5. まったくない

問７-30　他の市町に比べて、あなたがお住まいだった市町の被害はどの程度だったと思いますか。（○はひとつ）

1. 甚大だったと思う
2. やや甚大だったと思う
3. 同等だったと思う
4. やや小さかったと思う
5. かなり小さかったと思う

問７-31　義援金や支援金の程度はいかがですか。（○はひとつ）

1. とても満足
2. やや満足
3. どちらでもない
4. やや不満
5. とても不満

【住まいの再建を検討されている、もしくは再建をすでにされた方のみお答えください】

問７-32　住まいの再建のための資金は確保できていますか。（○はひとつ）

1. 十分確保できる・できた
2. まあまあ確保できる・できた
3. やや不足している・した
4. まったく足りない

問７-33　震災前に地震保険に加入していましたか。（○はひとつ）

1. 加入していて，支弁があった
2. 加入していたが，支弁はなかった
3. 加入していなかった

問７-34　現在、借金・ローンはありますか。（○はひとつ）

1. ない
2. ある（返済の目処がおおむねたっている）
3. ある（返済の目処がたっていない）

問７-35　現在のご家族の精神状態は落ち着いていますか。（○はひとつ）

1. 落ち着いている
2. 多少問題がある
3. 大きな問題がある

問７-36　今後の生活再建のプランについて家庭内で意見は一致していますか。（○はひとつ）

1. 一致している
2. おおむね一致している
3. あまり一致していない
4. 完全に意見が分かれている
5. 検討していない

5 ページでは地震発生以前のご自身の状況をおたずねしました。
ここでは現在のあなたの状況についておたずねします。

問８－１　現在、身体の状態はいかがですか。（○はひとつ）

健康である　　　　　　　　　　　　　　　非常に悪い
0 ―― 1 ―― 2 ―― 3 ―― 4 ―― 5

問８－２　現在、心の状態はいかがですか。（○はひとつ）

健康である　　　　　　　　　　　　　　　非常に悪い
0 ―― 1 ―― 2 ―― 3 ―― 4 ―― 5

問８－３　現在、運動の習慣はありますか。（○はひとつ）

1. ほぼ毎日　2. ときどき　3. ほとんどない　4. 全くない

問８－４　現在、飲酒の習慣はありますか。（○はひとつ）

1. ほぼ毎日　2. ときどき　3. ほとんどない　4. 全くない

問８－５　現在、経済的な不安はありますか。（○はひとつ）

ない　　　　　　　　　　　　　　　　　　非常に強い
0 ―― 1 ―― 2 ―― 3 ―― 4 ―― 5

問８－６　現在、家族との関係はどうですか。（○はひとつ）

1. 良い　2. やや良い　3. やや悪い
4. 悪い　5. 家族はいない

問８－７　現在、親族との関係はどうですか。（○はひとつ）

1. 良い　2. やや良い　3. やや悪い
4. 悪い　5. 親族はいない

問８－８　現在、友人との関係はどうですか。（○はひとつ）

1. 良い　2. やや良い　3. やや悪い
4. 悪い　5. 友人はいない

問８－９　現在、職場の人間関係はどうですか。（○はひとつ）

1. 良い　2. やや良い　3. やや悪い
4. 悪い　5. 仕事はしていない

問８－10　現在、近所にいる友人・知人はどのくらいですか。（○はひとつ）

1. とても多い　2. やや多い　3. 少ない　4. いない

問８－11　現在、世帯での自家用車の保有台数は何台ですか。（○はひとつ）

1. ０台　2. １台　3. ２台以上

最後に、ご回答いただいた方ご自身について、いくつかおたずねします。調査結果を統計的に解析するため必要な事柄ですので、ご回答をよろしくお願いいたします。

Ｆ１　あなたの性別をお答えください。

1. 男　性	2. 女　性

Ｆ２　あなたの年齢をお答えください。

1. 20～29 歳	2. 30～39 歳
3. 40～49 歳	4. 50～59 歳
5. 60～69 歳	6. 70～79 歳
7. 80 歳以上	

Ｆ３　あなたが最後に卒業された学校（中退、在学中を含む）を以下の中からお答えください。

1. 中学校（旧制小学校・旧制高等小学校）	2. 高等学校（旧制中学校・女学校）
3. 高等専門学校・短期大学（旧制高等学校）	4. 大学・大学院
5. その他（　　　　　　　　　　）	

以下の項目については震災前後両方についてお答えください。

Ｆ４　あなたの職業を教えてください。
二つ以上の職業を持っている場合は主な収入源になっている職業をお答えください。（○はひとつ）

**震災前**	1. 農林漁業者・自営業者（家族従事者を含む）	2. 経営者
	3. 勤め人（管理職を含む）	4. 家事専業
	5. 学生	6. 無職
	7. その他（　　　　　　）	
**震災後**	1. 農林漁業者・自営業者（家族従事者を含む）	2. 経営者
	3. 勤め人（管理職を含む）	4. 家事専業
	5. 学生	6. 無職
	7. その他（　　　　　　）	

Ｆ５　あなたの世帯年収はどのぐらいですか。（○はひとつ）

**震災前**	1. 200 万円未満	2. 200～399 万円
	3. 400～599 万円	4. 600～799 万円
	5. 800～999 万円	6. 1000～1199 万円
	7. 1200～1399 万円	8. 1400 万円以上
**震災後**	1. 200 万円未満	2. 200～399 万円
	3. 400～599 万円	4. 600～799 万円
	5. 800～999 万円	6. 1000～1199 万円
	7. 1200～1399 万円	8. 1400 万円以上

Ｆ６　あなたの同居家族の世帯構成をお選びください。（○はひとつ）

**震災前**	1.　単身世帯（おひとりでお住まい）	2.　夫婦のみ世帯
	3.　二世代世帯（親子で同居）	4.　三世代世帯（親・子・孫で同居）
	5.　その他（　　　　　　　　　）	
**震災後**	1.　単身世帯（おひとりでお住まい）	2.　夫婦のみ世帯
	3.　二世代世帯（親子で同居）	4.　三世代世帯（親・子・孫で同居）
	5.　その他（　　　　　　　　　）	

Ｆ７　あなたの同居家族の中に日常生活に介助や介護が必要な方はいますか。（○はひとつ）

**震災前**	1.　はい	2.　いいえ
**震災後**	1.　はい	2.　いいえ

Ｆ８－１　あなたの居住形態をお答えください。（○はひとつ）

**震災前**	1.　持ち家	2.　社宅・官舎等
	3.　公営住宅	4.　家族・親せき・知人宅
	5.　民間賃貸住宅	6.　その他（　　　　　　　　　）
**震災後**	1.　震災前と同じ持ち家	2.　震災後に新築・購入した持ち家
	3.　社宅・官舎等	4.　仮設住宅
	5.　民間賃貸みなし仮設住宅	6.　公営住宅
	7.　家族・親せき・知人宅	8.　みなし仮設以外の民間賃貸住宅
	9.　その他（　　　　　　　　　）	

Ｆ８－２　上記、現在の震災後のお住まいに至る間に、指定避難所や親族･友人などの家に身を寄せていた等の避難生活を経験されましたか。（○はひとつ）

1.　避難生活を経験した	2.　避難生活は経験しなかった

☆最後に、このアンケートへのご回答（ご記入）にかかったおよその時間を教えてください。
（休憩や中断をはさんだ方は、その時間を除いて実際に記入に要した時間でお答えください）

およそ（　　　）時間（　　　）分

－　アンケートは以上です。ご協力ありがとうございました　－

分析結果をご希望の方は、以下にご記入ください！

ご回答の内容に基づいた、あなたの「性格・考え方・習慣」と震災での経験の関係について、分析結果を発送いたします。

分析結果の返送をご希望の方には、下欄に返送先など必要事項をご記入の上ご返送ください。（平成２６年３月末頃、発送の予定です）

氏　名		電話番号	
住　所	〒　　　　－		

本欄は、分析結果発送のためのみで使用します。
回答内容は、無記名で集計しますのでご安心ください。
