# Supplementary material for: Self-help and mutual assistance in the aftermath of a tsunami: How individual factors contribute to resolving difficulties
Source: PLoS One. 2021 Oct 7;16(10):e0258325. doi: 10.1371/journal.pone.0258325 (PMC8496872; doi:10.1371/journal.pone.0258325)
Supplement: Attachment — Submitted filename: Review Evaluation Report-PONE-D-20-29963-13.02.021.doc [file pone.0258325.s004.doc]

**Paper title:** Policy liberalism 1 and source of news predict pandemic-related health behaviors and trust in the scientific community

**Received date:** 05/02/2021	**Aim(s):** Peer Review

**DOI/ID:** PONE-D-20-29963	**Review due date:** 2021 /02 /15

| **Section** | **Review comments and notes** |
| --- | --- |
| Abstract, title and references | - The manuscript title is relevant and sufficient informative,  -Abstract; the author well elucidated the study background, objectives and methods  -Keywords are well presented  -The abstract need to be concise  -the study aim is not clear  -Data collection time acquisition needs to mention  -The authors are request to add implication of the study  -Few references are not appropriately listed  - Many references are not recent.  -the authors are appropriately key studies included |
| Introduction/  background | -the author may add few points about disaster management cycle, it will help to understand the overall concept of disaster to readers.  -Need to define vulnerability and aftermath/after disaster what kind of vulnerability has to face of people.  -The authors are request to add research gap and innovation of the study  -The introduction part appropriately elucidated the study aims, objectives, problems and its important. But still need to work on research gap, innovation, implication part.  -What is dependent variable? |
| Methods | - The **Table 1.** Damage and demographic profiles of the participants.; need to concise  -**Table 2.** Experiences of difficulty and 166 how they were resolved. Can be put in supplementary part.  -**Table 3.** Personality factors. Need to put in supplementary section.  -the authors used a scale to evaluate the degree to participants had experienced difficulty; scale 0= none, 1= Little and 2= very high. Is this scale used previous any studies? If yes, can you please add citation?  -Is there any basis to divided into clusters?  - Line no.-249; need ref.?  The methodology part needs to concise rather details presentation and explanation. |
| Results | -Line no.-308; in Cluster-2; what are the privacy item? Can you add these?  -Line no.-370; need Ref.?  -Results part may concise |
| Discussion and Conclusions | -what is relationship difficulty with “body temperature regulation” is there any citation?  -Line no-511, the income level didn’t affect the success in any resolution; it is not clear, because pro-survival and post disaster, the individual and country level there is an emergency, so how income level didn’t affect the resolution.  -Line no.-540, where is vulnerability indices?  -Line no-570; need citation.  -the authors are advice to avoid unnecessary discussion because it creates lengthy discussion.  -The authors are advice to discuss the main issue-based analysis what they found.  -Conclusion need to be concise, need to focus on key findings based on analysis |
| Overall | I read the paper carefully and I wish to support it for publication after minor revision. This is an original work and depth of research is accurate. The study has significance impact on wide range of researcher in this field of disaster management. The manuscript has been written in Standard English. The research question is well defined. In my eyes, the main comments of the paper are as follows:  1) Introduction part, the author may add few points about disaster management cycle, it will help to understand the overall concept of disaster to readers. The authors are request to add research gap and innovation of the study.  2) The methodology part needs to concise rather details presentation and explanation.  3) Results part may concise  4) Conclusions part need to include key findings of the study.  Overall, the paper should need minor revision. |
