# Supplementary material for: Self-help and mutual assistance in the aftermath of a tsunami: How individual factors contribute to resolving difficulties
Source: PLoS One. 2021 Oct 7;16(10):e0258325. doi: 10.1371/journal.pone.0258325 (PMC8496872; doi:10.1371/journal.pone.0258325)
Supplement: Attachment — Submitted filename: response_letter_R1.docx [file pone.0258325.s005.docx]

**Response to Reviewer #1:**

*C1.1) Abstract and title*

*C1.1.1) The abstract need to be concise*

R1.1.1) To make the abstract concise, we reduced and reorganized the report of the results as follows (L26–35):

“We identified general life difficulties and medico-psychological difficulties as two broad types of difficulty; disaster damage contributed to both types, while some personality factors (e.g., neuroticism) exacerbated the latter. Disaster damage hampered self-resolution and forced a reliance on resolution through cooperation or public assistance. On the other hand, some demographic factors, such as being young and living in a three-generation household, facilitated resolution thorough the family. Several personality factors facilitated different types of resolution, primarily of general life difficulties; the problem-solving factor facilitated self-resolution, altruism, or stubbornness resolutions through requests, leadership resolution through acquaintance, and emotion-regulation resolution through public assistance.”

*C1.1.2) The study aim is not clear*

R1.1.2) We clarified our study aim as follows (L19–21):

“To identify such individual factors in the background (i.e., disaster damage and demographic) and personality domains considering different types of difficulty and resolution, we analyzed…”

*C1.1.3) Data collection time acquisition needs to mention*

R1.1.3) The time of data collection is now mentioned as follows (L21–22):

“we analyzed survey data collected in the 3-year aftermath of the 2011 Great East Japan Earthquake and Tsunami.”

*C1.1.4) The authors are request to add implication of the study*

R1.1.4) The implications of the study are now clarified as follows (L35–39):

“Our findings are the first to demonstrate the involvement of different individual, particularly personality, factors in survival in the complex social dynamics of this disaster stage. They may contribute to disaster risk mitigation, allowing sophisticated risk evaluation and community resilience building.”

*C1.2) Introduction*

*C1.2.0) the author may add few points about disaster management cycle, it will help to understand the overall concept of disaster to readers. The authors are request to add research gap and innovation of the study.*

R1.2.0) We thank the reviewer for this advice. We included the disaster management cycle perspective and described the significance of our study in this context as follows:

(L47–50)

“Among the stages of the disaster management cycle (i.e., mitigation, preparation, emergency, and recovery stages), the emergency stage is a critical period for intervention by organizations [4], but such public support takes time and is often limited.”

(L52-54)

“Understanding the individual factors that contribute to successfully resolving difficulties encountered is important for risk evaluation and for fostering citizens’ resilience during times of quiescence, namely, the mitigation stage [4].”

We also clarified gaps in the research in the second and third paragraphs and innovation in the fourth paragraph as follows:

Gap 1: paragraph 2 (L57–58)

“Published research on such individual factors has primarily addressed factors that contribute to negative consequences, that is, exacerbating factors.”

Gap 2: paragraph 3 (L73-86)

“To explore the individual factors that contribute to the resolution of difficulties, the following three issues should be addressed. First, different categories of difficulty may be dealt with separately given the apparently distinct characteristics of victims as represented by the social science and mental health fields... Second, different types of resolution should be addressed separately, as individual factors likely affect the availability of different resolution types… Finally, in addition to common background factors, we were interested in personality traits as individual factors...”

Innovation: paragraph 4 (L87-89)

“In this study, we investigated the individual background and personality factors that facilitate self-aid or mutual assistance in the immediate aftermath of a disaster considering different types of difficulty and resolution.”

*C1.2.1) Need to define vulnerability and aftermath/after disaster what kind of vulnerability has to face of people.*

R1.2.1) We recognize that the term “vulnerability” was used inappropriately in the original version of our manuscript to imply any factors that contribute to negative consequences (e.g., disaster damage). In the revised version, we limited the use of this term to factors that are not typically considered direct causes of the consequence, a distinction made elsewhere in the literature (e.g., 9–11). We now use the term “exacerbating factor” when referring to all factors that contribute to negative consequences.

*C1.2.2) What is dependent variable?*

R1.2.2) For the multiple regression analyses, the degree of difficulty experienced was the dependent variable used to identify exacerbating factors for each difficulty type, and the degree of successful resolution by each of the six resolution types was the dependent variable used to identify the factors facilitating the resolution of a difficulty. These are now clarified as follows:

(L212–214)

“We performed a three-block hierarchical multiple regression analysis [24] on the degree of difficulty experienced (i.e., entered as a dependent variable) separately for each identified difficulty type.”

(L252-255)

“We performed a hierarchical multiple regression analysis on the degree of successful resolution (i.e., entered as a dependent variable) separately for each of the six resolution types (i.e., self, request, family, acquaintance, cooperation, and public) for each difficulty type.”

*C1.3) Methods*

*C1.3.0) The methodology part needs to concise rather details presentation and explanation.*

R1.3.0) The Materials and Methods section was made more concise by moving Tables 2 and 3 in the original manuscript to the supplementary section (S1 and S2 Tables in S1 Results) and removing the explanation of the general structure of the analysis, which was already presented in the Introduction.

*C1.3.1) Table 1. Damage and demographic profiles of the participants.; need to concise*

R1.3.1) Table 1 was totally reorganized (L130). We removed the actual text of the questionnaire (the original survey is available in the supplementary materials) and put the variable labels, options, and statistics in different columns.

*C1.3.2) Table 2. Experiences of difficulty and 166 how they were resolved. Can be put in supplementary part.*

R1.3.2) We moved the original Table 2 to the supplementary section (S1 Table in S1 Results).

*C1.3.3) Table 3. Personality factors. Need to put in supplementary section.*

R1.3.3) We moved the original Table 3 to the supplementary section (S2 Table in S1 Results).

*C1.3.4) the authors used a scale to evaluate the degree to participants had experienced difficulty; scale 0= none, 1= Little and 2= very high. Is this scale used previous any studies? If yes, can you please add citation?*

R1.3.4) The scale was adopted without reference to any previous literature, not an uncommon practice in surveys. Here, a three-point scale, rather than a binary scale, was employed to encourage responders with a moderate degree of difficulty to select 1, as they would likely select “no” on a binary scale considering other seriously damaged victims. This motivation for adopting the three-point scale is now included as follows (L143–146):

“This was meant to encourage responders with a moderate degree of difficulty to select 1, as they would likely select “none” on a binary scale to differentiate their losses from those of seriously damaged victims.”

*C1.3.5) Is there any basis to divided into clusters?*

R1.3.5) In the Introduction, we have clarified the background and motivation for using cluster analysis to identify major types of difficulty:

Background (L74–78)

“First, different categories of difficulty may be dealt with separately given the apparently distinct characteristics of victims as represented by the social science and mental health fields. We were curious as to whether the distinction reflects a lack of integrated research between the two fields [15] or whether there are indeed different categories of difficulty.”

Motivation (L99–100)

“First, we explored the major types of difficulty among the 18 areas using a cluster analysis”

*C1.3.6) Line no.-249; need ref.?*

R1.3.6) We added a reference for the Bonferroni correction, along with an explanation, as follows (L216–219):

“The statistical threshold for exploratory analyses was set at p < 0.05, corrected for multiple comparisons using the Bonferroni method (adjusted p-value = raw p-value × number of tests [25]) based on the number of independent variables entered in the analyses.”

25.	Armstrong RA. When to use the Bonferroni correction. Ophthalmic Physiol Opt. 2014;34(5):502-8. doi: 10.1111/opo.12131.

*C1.4) Results*

*C1.4.0) Results part may concise*

R1.4.0) The Results section was made more concise by focusing the discussion of the hierarchical multiple regressions results on the summary tables (now Tables 2 and 3) and moving the original Tables 5–18 to the supplementary section (S3–16 Tables in S1 Results).

*C1.4.1) Line no.-308; in Cluster-2; what are the privacy item? Can you add these?*

R1.4.1) We apologize for the misleading wording. We were referring to the 18th type of experienced difficulty, as described in the Variables section of the Methods. For clarity, we updated the description as follows (L274-276):

“Cluster 2 had privacy at its center and medical care for oneself and family members, individual and family psychological stress, noise, stench, appropriate spare clothes, and sleeping at the periphery.”

*C1.4.2) Line no.-370; need Ref.?*

R1.4.3) As a reference for a hierarchical multiple regression analysis including the stepwise selection of variables, we have cited a textbook (R212–216):

“We performed a three-block hierarchical multiple regression analysis [24] on the degree of difficulty experienced (i.e., entered as a dependent variable) separately for each identified difficulty type. First, we explored background factors using a stepwise approach in blocks 1 and 2. Then, we determined whether each personality factor had additional explanatory power in block 3.”

24.	Tabachnick BG, Fidell LS. Multiple Regression. Using Multivariate Statistics. 7th ed. Boston: Pearson Education,Inc.; 2007. p. 99-166.

*C1.5) Discussion and Conclusions*

*C1.5.0) Conclusions part need to include key findings of the study.*

R1.5.0) We summarized our key findings in the Conclusion as follows (L491–506).

“…We first identified two types of difficulties: general life difficulties, which were experienced by most victims, and medico-psychological difficulties, which were experienced to varying degrees across victims. Both types were exacerbated by disaster damages, whereas medico-psychological difficulties were uniquely affected by personality factors, with neuroticism and extraversion acting as exacerbating and mitigating factors, respectively.

Then we examined the effect of individual factors on success in resolving the two types of difficulty through six types of resolution: self-resolution, four types of mutual assistance, and public assistance. In general, disaster damage hampered self-resolution and forced a reliance on cooperation or public assistance. Some demographic factors, such as being young and living in a three-generation household, facilitated resolution thorough the family. Importantly, several personality factors facilitated different types of resolution, primarily of general life difficulties: problem solving facilitated self-resolution, altruism or stubbornness facilitated resolution through request, leadership facilitated resolution through acquaintances, and emotion regulation facilitated resolution through public assistance.”

*C1.5.1) What is relationship difficulty with “body temperature regulation” is there any citation?*

R1.5.1) This is an interpretation of our results, specifically, the item “room temperature” in cluster 1. This is now clarified by including the relevant items in the discussion of results (L356–359):

“general difficulties in meeting basic life needs, such as food (eating and cooking), body temperature regulation (room temperature), and sanitation (toilet, washing one’s face, bathing, and laundry),”

*C1.5.2) Line no-511, the income level didn’t affect the success in any resolution; it is not clear, because pro-survival and post disaster, the individual and country level there is an emergency, so how income level didn’t affect the resolution.*

R1.5.2) The argument was based on our results showing that income was not identified as a mitigating factor for any difficulty type or a facilitative factor for any resolution type. This is now clarified as follows (L415–417):

“Contrary to the commonly held view that low socioeconomic status is a strong vulnerability factor, income was not identified as a mitigating factor of any difficulty type or as a facilitative factor for any resolution type.”

We discussed this because, as the reviewer pointed out, the finding is surprising. Our speculation regarding this negative finding is as follows (L421–423):

“Socioeconomic status may play little role in the immediate aftermath, where difficulties are mostly caused by the shortage of life-critical resources due to the disruption of logistical systems, which is not something that can be solved with money.”

*C1.5.3) Line no.-540, where is vulnerability indices?*

R1.5.3) The description no longer exists, as the paragraph has been removed.

*C1.5.4) Line no-570; need citation.*

R1.5.4) The argument was based on our own data (Table 1). It is now referenced as follows (L426–427):

“our victims were relatively well off (i.e., only 12% had yearly income less than 2 million JPY, or approximately 18,000 USD; Table 1)”

*C1.5.5) the authors are advice to avoid unnecessary discussion because it creates lengthy discussion.*

R1.5.5) We removed two paragraphs from the detailed interpretation of the contribution of each personality factor and a summary paragraph from the discussion.

*C1.5.6) The authors are advice to discuss the main issue-based analysis what they found.*

R1.5.6) After excluding a lengthy discussion of the personality factors (cf. R1.5.5), we believe our discussion is now focused on the findings. To call attention to the implications of our findings instead, we added the following discussion (L442–448):

“In the context of disaster risk mitigation focused on evaluating risk and fostering citizen resilience, each personality factor implicated in difficulty-exacerbating or resolution-facilitative approaches is theoretically a target for intervention. A classic approach attempted to include psycho-behavioral factors in explaining different levels of disaster risk between two population of residents in the context of tornado evacuation [31]. Inclusion of the personality factors identified in the current study might contribute to more sophisticated risk evaluation among citizens in the emergency stage.”

*C1.5.7) Conclusion need to be concise, need to focus on key findings based on analysis*

C1.5.7) We improved the conciseness of the Conclusions section by focusing on the key findings (cf. R1.5.0) and clarifying their implications as follows (L507–514):

“Our results demonstrate the importance of dissociating difficulties and resolution types when identifying facilitative factors for self-aid and mutual assistance in this disaster stage. The identified involvement of different individual factors, particularly personality factors, in different types of resolution may reflect the complexity of social dynamics as well as the psychological processes involved in survival in this context. The current findings contribute to the improvement of disaster risk mitigation through risk evaluation and the fostering of resilience among citizens by allowing separate consideration of different difficulty types and resolution processes.”

C1.6) References

C1.6.1) Few references are not appropriately listed

R1.6.1) We have carefully checked the reference list to assure that it conforms to the journal’s requirements.

*C1.6.2) Many references are not recent.*

R1.6.2) We checked the literature and added the following newer references in support of two of our arguments.

- Disaster mental-health literature on contributing individual factors:

14.	Goldmann E, Galea S. Mental health consequences of disasters. Annu Rev Public Health. 2014;35(1):169-83. doi: 10.1146/annurev-publhealth-032013-182435.

37.	Zoellner T, Maercker A. Posttraumatic growth in clinical psychology - a critical review and introduction of a two component model. Clin Psychol Rev. 2006;26(5):626-53. doi: 10.1016/j.cpr.2006.01.008.

Although several references for other issues are not recent, we believe that the more recent literature offers no fundamental updates to the earlier arguments.

**Response to Reviewer #2:**

*C2.1) Abstract*

*It is not well structured, even the essential points are not clear. It is relatively full of background text rather than specific point. The author is advised to revise the abstract by following the journal style (academic way).*

R2.1) We totally revised the abstract to clarify the background, purpose, and implications of the study, as follows (L17–39):

“Self-aid and mutual assistance among victims are critical for resolving difficulties in the immediate aftermath of a disaster, but individual facilitative factors for such resolution processes are poorly understood. To identify such individual factors in the background (i.e., disaster damage and demographic) and personality domains considering different types of difficulty and resolution, we analyzed survey data collected in the 3-year aftermath of the 2011 Great East Japan Earthquake and Tsunami. We first identified major types of difficulty using a cluster analysis of 18 difficulty domains and then explored individual factors that facilitated six types of resolution (self-help, request for help, help from family, help from an acquaintance, help through cooperation, and public assistance) of these difficulty types. We identified general life difficulties and medico-psychological difficulties as two broad types of difficulty; disaster damage contributed to both types, while some personality factors (e.g., neuroticism) exacerbated the latter. Disaster damage hampered self-resolution and forced a reliance on resolution through cooperation or public assistance. On the other hand, some demographic factors, such as being young and living in a three-generation household, facilitated resolution thorough the family. Several personality factors facilitated different types of resolution, primarily of general life difficulties; the problem-solving factor facilitated self-resolution, altruism, or stubbornness resolutions through requests, leadership resolution through acquaintance, and emotion-regulation resolution through public assistance. Our findings are the first to demonstrate the involvement of different individual, particularly personality, factors in survival in the complex social dynamics of this disaster stage. They may contribute to disaster risk mitigation, allowing sophisticated risk evaluation and community resilience building.”

*C2.2) Introduction*

*The introduction is not well-organized, and it has been unnecessarily illustrated with irrelevant text. The authors missed up this section's sequence, and also, the rationale of this study is not well placed. The authors failed to present the core issue and its importance. The author is advised to concentrate on the main issue, significance of this study, avoid unnecessary discussions, and write in a logical order.*

R2.2) We totally reorganized the Introduction in the standard logical structure of a scientific paper. We particularly emphasized the clarification of research gaps and innovations:

Gap 1: paragraph 2 (L57–58)

“Published research on such individual factors has primarily addressed factors that contribute to negative consequences, that is, exacerbating factors.”

Gap 2: paragraph 3 (L73-86)

“To explore the individual factors that contribute to the resolution of difficulties, the following three issues should be addressed. First, different categories of difficulty may be dealt with separately given the apparently distinct characteristics of victims as represented by the social science and mental health fields... Second, different types of resolution should be addressed separately, as individual factors likely affect the availability of different resolution types… Finally, in addition to common background factors, we were interested in personality traits as individual factors...”

Innovation: paragraph 4 (L87-89)

“In this study, we investigated the individual background and personality factors that facilitate self-aid or mutual assistance in the immediate aftermath of a disaster considering different types of difficulty and resolution.”

*C2.3) Materials and Method*

*This section is very weak. It requires revised by following essential scientific steps.*

*Table-1, 2 can be sorted in accordance with some key aspects like category, questions, coding, frequency and so on; simultaneously the description should be scientific and coherent.*

R2.3) The Materials and Methods section was revised to focus on the essential logical steps; we moved the original Tables 2 and 3 to the supplementary section (S1 and S2 Tables in S1 Results). We also removed our explanation of the general structure of the analysis, which was already noted in the Introduction. Table 1 was totally reorganized (L130). We removed the actual text of the questionnaire (which is available through the original survey in the supplementary section) and put the variable labels, options, and statistics in different columns.

*C2.4) Results and discussion*

*C2.4.1) Discussion- this part is more descriptive than the explanation of the results. The author should take appropriate and well-organized measures to clarify the study's findings.*

R2.4.1) The summary of results in the initial paragraph was made more concise; we did not completely remove it in accordance with the suggestion of Reviewer #1. We believe that the rest of the discussion goes beyond mere explanation and addresses the significance or implications of the findings. We also excluded the lengthy discussion of personality factors. We believe that our discussion is now focused on our findings.

*C2.4.2) Conclusion*

*It should be revised and followed the updated version*

R2.4.2) The Conclusion was totally rewritten based on the updated Discussion (L488–514):

“Analyzing the data from the 2011 Great East Japan Earthquake and Tsunami Disaster, we explored individual background and personality factors that facilitate self-aid or mutual assistance in the immediate aftermath of a disaster considering different types of difficulty and resolution. We first identified two types of difficulties: general life difficulties, which were experienced by most victims, and medico-psychological difficulties, which were experienced to varying degrees across victims. Both types were exacerbated by disaster damages, whereas medico-psychological difficulties were uniquely affected by personality factors, with neuroticism and extraversion acting as exacerbating and mitigating factors, respectively.

Then we examined the effect of individual factors on success in resolving the two types of difficulty through six types of resolution: self-resolution, four types of mutual assistance, and public assistance. In general, disaster damage hampered self-resolution and forced a reliance on cooperation or public assistance. Some demographic factors, such as being young and living in a three-generation household, facilitated resolution thorough the family. Importantly, several personality factors facilitated different types of resolution, primarily of general life difficulties: problem solving facilitated self-resolution, altruism or stubbornness facilitated resolution through request, leadership facilitated resolution through acquaintances, and emotion regulation facilitated resolution through public assistance.

Our results demonstrate the importance of dissociating difficulties and resolution types when identifying facilitative factors for self-aid and mutual assistance in this disaster stage. The identified involvement of different individual factors, particularly personality factors, in different types of resolution may reflect the complexity of social dynamics as well as the psychological processes involved in survival in this context. The current findings contribute to the improvement of disaster risk mitigation through risk evaluation and the fostering of resilience among citizens by allowing separate consideration of different difficulty types and resolution processes.”

*C2.5) References*

*You should critically look at your references like capital letter, small letter, issue, etc.*

R2.5) We have carefully checked the reference list to assure that it conforms to the journal’s requirements.

**Response to Journal Requirements**

*C3.1) Please ensure that your manuscript meets PLOS ONE's style requirements, including those for file naming. The PLOS ONE style templates can be found at*

*https://journals.plos.org/plosone/s/file?id=wjVg/PLOSOne_formatting_sample_main_body.pdf and*

*https://journals.plos.org/plosone/s/file?id=ba62/PLOSOne_formatting_sample_title_authors_affiliations.pdf*

R3.1) We made sure that our revised manuscript meets these requirements before submission.

*C3.2) Please include a copy of Table 12 which you refer to in your text on page 29.*

R3.2) We apologize for mislabeling this table. It is now included in the supplementary materials (S1 Results) with the label “S10 Table.”
