# Supplementary material for: Self-help and mutual assistance in the aftermath of a tsunami: How individual factors contribute to resolving difficulties
Source: PLoS One. 2021 Oct 7;16(10):e0258325. doi: 10.1371/journal.pone.0258325 (PMC8496872; doi:10.1371/journal.pone.0258325)
Supplement: Attachment — Submitted filename: 3R1-Re-Review Report-PONE-D-20-29963R1-22.06.2021-R1.doc [file pone.0258325.s006.doc]

**Paper title:** Self-help and mutual assistance in the aftermath of a tsunami: how individual factors contribute to resolving difficulties

**Received date: 11/06/2021	Aim(s):** **Re-**Review

DOI/ID: **PONE-D-20-29963R1**	Review due date: 2021 /06 /22

| **Section** | **Re-Review comments** |
| --- | --- |
| Abstract, title  and references | -The authors have reduced, reorganized and concise the abstract properly.  -they clarified the study aim in the revised version.  -the authors have mentioned the data collection time acquisition time.  -The authors have rewritten the implication of the research findings properly |
| Introduction/ Background | -The authors have added some text about disaster management cycle and written research gap with innovation part properly.  -the author properly clarified the issues of aftermath/after disaster what kind of  vulnerability has to face of people.  -they used multiple regression analysis. |
| Methods | -The authors have revised the The Materials and Methods section and concise by moving Tables 2 and 3 in the original manuscript to the supplementary section  -they addressed other issues raised by reviewer |
| Results | -The authors have concise this part according to the reviewer suggestion.  -they have addressed other issues marked by reviewer |
| Discussion and Conclusions | -the author has revised and concise the section based on reviewer comments.  -the authors read carefully all other comments and revised the manuscript accordingly.  -They have updated the reference section and followed the Journal guidelines. |
| Overall | I’ve read the author responses/revision and the revised manuscript whether they have addressed the issues properly or not. Based on my understanding the authors have addressed all the comments and revised their manuscript appropriately.  Overall, the paper may consider for publication after minor revision. |
