# Supplementary material for: Self-help and mutual assistance in the aftermath of a tsunami: How individual factors contribute to resolving difficulties
Source: PLoS One. 2021 Oct 7;16(10):e0258325. doi: 10.1371/journal.pone.0258325 (PMC8496872; doi:10.1371/journal.pone.0258325)
Supplement: Attachment — Submitted filename: response_letter_R2.docx [file pone.0258325.s007.docx]

**Response to Reviewer #1:**

*C1.1) Abstract, title and references*

*-The authors have reduced, reorganized and concise the abstract properly.*

*-they clarified the study aim in the revised version.*

*-the authors have mentioned the data collection time acquisition time.*

*-The authors have rewritten the implication of the research findings properly*

*C1.2) Introduction/ Background*

*-The authors have added some text about disaster management cycle and written research gap with innovation part properly.*

*-the author properly clarified the issues of aftermath/after disaster what kind of vulnerability has to face of people.*

*-they used multiple regression analysis.*

*C1.3) Methods*

*-The authors have revised the The Materials and Methods section and concise by moving Tables 2 and 3 in the original manuscript to the supplementary section*

*-they addressed other issues raised by reviewer*

*C1.4) Results*

*-The authors have concise this part according to the reviewer suggestion.*

*-they have addressed other issues marked by reviewer*

*C1.5) Discussion and Conclusions*

*-the author has revised and concise the section based on reviewer comments.*

*-the authors read carefully all other comments and revised the manuscript accordingly.*

*-They have updated the reference section and followed the Journal guidelines.*

*C1.6) Overall*

*I’ve read the author responses/revision and the revised manuscript whether they have addressed the issues properly or not. Based on my understanding the authors have addressed all the comments and revised their manuscript appropriately.*

*C1.7) Overall, the paper may consider for publication after minor revision.*

R1) We are happy that we could respond appropriately to all the comments from this reviewer. We thank them again for their constructive comments. While C1.7 may be read as there is something left for minor revision, we understand that it is meant for the points raised by other reviewers since no specific issues are raised by this reviewer.

**Response to Reviewer #2:**

*C2) I looked at the manuscript carefully and I found that the author responded appropriately to each of the raised questions. I hope that it is now suitable to be published..*

R2) We are very pleased to be able to respond appropriately to all the comments from this reviewer. We thank them again for their constructive comments.

**Response to Reviewer #3:**

*C3.0) The study examines the relationship between individuals’ contributions to the tsunami effects. While the paper offers an interesting understanding, it needs more work to be published.*

R3.0) We thank the reviewer for their constructive suggestions. We suppose these are based on the first version of our manuscript (ver. 1). We believe that we have already addressed many of the issues raised in this review in the first extensive revision (ver.2), which addressed a number of comments from the two reviewers. However, some issues were newly pointed out, and we have made new responses to them in this revised version (ver.3). In the revised version (ver. 3) including the revision history, the first revision corresponding to the issues pointed out this time is indicated in blue letters, and the new revision this time is indicated in red letters.

*C3.1) Abstract:*

*C3.1.1) The abstract needs a smooth transition through the tsunami and individual aspects. In the current edition, there is an abrupt jump to the study without a clear concentration.*

R3.1.1) In the first sentence of the abstract, by focusing on the core issues only, we made a smooth transition from the academic background to our research motivation as follows (L17–19):

“Self-aid and mutual assistance among victims are critical for resolving difficulties in the immediate aftermath of a disaster, but individual facilitative factors for such resolution processes are poorly understood.”

*C3.1.2) It has a lot of irrelevant information that should not be here. Just mention the study aim and key findings, not the whole story and every single finding or discussion here.*

R3.1.2) We now clarified the research purpose and removed some descriptions that might have disturbed readers’ understanding. While we could not diminish the results given the exploratory nature of the study and the study’s significance in terms of the complexity of the results, we believe that such an intention is now clear in the revised abstract as follows (L19–37):

“To identify such individual factors in the background (i.e., disaster damage and demographic) and personality domains considering different types of difficulty and resolution, we analyzed survey data collected in the 3-year aftermath of the 2011 Great East Japan Earthquake and Tsunami. We first identified major types of difficulty using a cluster analysis of 18 difficulty domains and then explored individual factors that facilitated six types of resolution (self-help, request for help, help from family, help from an acquaintance, help through cooperation, and public assistance) of these difficulty types. We identified general life difficulties and medico-psychological difficulties as two broad types of difficulty; disaster damage contributed to both types, while some personality factors (e.g., neuroticism) exacerbated the latter. Disaster damage hampered self-resolution and forced a reliance on resolution through cooperation or public assistance. On the other hand, some demographic factors, such as being young and living in a three-generation household, facilitated resolution thorough the family. Several personality factors facilitated different types of resolution, primarily of general life difficulties; the problem-solving factor facilitated self-resolution, altruism, or stubbornness resolutions through requests, leadership resolution through acquaintance, and emotion-regulation resolution through public assistance. Our findings are the first to demonstrate the involvement of different individual, particularly personality, factors in survival in the complex social dynamics of this disaster stage.”

*C3.2) Introduction:*

*C3.2.1) What is self-aid and mutual assistance? The reader needs a great explanation.*

R3.2.1) We clarified the context of the self-aid and mutual assistance addressed in this study by additionally explaining their roles in the disaster management cycle as follows (L45–51):

“Victims of natural disasters experience a variety of difficulties in the immediate aftermath, such as access to food, water, sanitation, and medical assistance, as well as psychological problems [1-3]. Among the stages of the disaster management cycle (i.e., mitigation, preparation, emergency, and recovery stages), the emergency stage is a critical period for intervention by organizations [4], but such public support takes time and is often limited. Self-aid and mutual assistance among victims in resolving such difficulties are critical for survival before public support becomes available [5-7].”

*C3.2.2) What are the individuals factors the author(s) believe studied poor? Also, it is so early to start what is not “studied well” in this section. Mention what the key missing parts by providing some “studied” aspects.*

R3.2.2) We reorganized the relevant paragraphs. The new second paragraph is now dedicated to the review of what has previously been studied as follows (L57–72):

“Published research on such individual factors has primarily addressed factors that contribute to negative consequences, that is, exacerbating factors. In this regard, the social science and mental health literatures have provided rather different images of victims. In the social science literature, the image of victims is rather vague in terms of the difficulties they encounter. Proposals of vulnerability factors [9-11] have been based on reviews of studies that describe vulnerability in association with various outcomes throughout the disaster stages, including casualty, physical or mental health, post-disaster socioeconomic states, and disaster preparedness. They commonly point out low socioeconomic status as a significant vulnerability factor. On the other hand, the image of the victim is clear in the mental health literature, primarily addressing poor mental health outcomes, such as post-traumatic stress disorders (PTSD). Although various individual factors have also been identified as risk factors, robust examination of disaster exposure and psychosocial resources (e.g., psychiatric pre-morbidity and personality) seems to be unique to the mental health literature [12-14]. In both literatures, the contributions of several common individual factors such as age, sex, and education are controversial.”

Then we sorted out the poorly studied aspects of the individual factors in the third paragraph as follows (L73–86):

“To explore the individual factors that contribute to the resolution of difficulties, the following three issues should be addressed. First, different types of difficulty may be dealt with separately given the apparently distinct characteristics of victims as represented by the social science and mental health fields. We were curious as to whether the distinction reflects a lack of integrated research between the two fields [15] or whether there are indeed different types of difficulty. Second, different types of resolution should be addressed separately, as individual factors likely affect the availability of different resolution types. For example, self-help may be limited by disaster damage, mutual assistance between familiar people may depend on existing social capital, and that between unfamiliar people may require an explicit request or proposal [16, 17]. Finally, in addition to common background factors, we were interested in personality traits as individual factors. Understanding personality traits, or individual psycho–behavioral characteristics, may be a promising approach for educational or cultural investigations into improving resilience [7, 11].”

*C3.2.3) p.5: 18 areas or 18 categories?*

R3.2.3) We meant 18 ‘areas’ when we address different items of difficulty in the original survey. The term ‘category’ in the original manuscript was meant for a group of such difficulty items that share some attribute; we used ‘types’ for this term in the revised manuscript. We hope that the meanings of these two terms are now clear in the revised manuscript.

*C3.2.4) Can you please refine the core study aims of the research as it seems there is no clear concentration? Please, highlight the study contributions clearly.*

R3.2.4) We clarified the core study aims of the research in the fourth paragraph of the revised Introduction as follows (L87–89):

“In this study, we investigated the individual background and personality factors that facilitate self-aid or mutual assistance in the immediate aftermath of a disaster considering different types of difficulty and resolution.”

This part is preceded by a paragraph where our research questions are sorted into three issues accompanied by the significance of their clarification as follows (L73–86; c.f., R3.2.2):

“To explore the individual factors that contribute to the resolution of difficulties, the following three issues should be addressed. First, different types of difficulty may be dealt with separately given the apparently distinct characteristics of victims as represented by the social science and mental health fields. We were curious as to whether the distinction reflects a lack of integrated research between the two fields [15] or whether there are indeed different types of difficulty. Second, different types of resolution should be addressed separately, as individual factors likely affect the availability of different resolution types. For example, self-help may be limited by disaster damage, mutual assistance between familiar people may depend on existing social capital, and that between unfamiliar people may require an explicit request or proposal [16, 17]. Finally, in addition to common background factors, we were interested in personality traits as individual factors. Understanding personality traits, or individual psycho–behavioral characteristics, may be a promising approach for educational or cultural investigations into improving resilience [7, 11].”

*C3.2.5) I have not seen any literature review section!? I am pretty sure there are some studies to be covered.*

R3.2.5) We believe our new second paragraph now makes the literature review of what has previously been studied on individual factors as follows (L57–72; c.f., R3.2.2):

“Published research on such individual factors has primarily addressed factors that contribute to negative consequences, that is, exacerbating factors. In this regard, the social science and mental health literatures have provided rather different images of victims. In the social science literature, the image of victims is rather vague in terms of the difficulties they encounter. Proposals of vulnerability factors [9-11] have been based on reviews of studies that describe vulnerability in association with various outcomes throughout the disaster stages, including casualty, physical or mental health, post-disaster socioeconomic states, and disaster preparedness. They commonly point out low socioeconomic status as a significant vulnerability factor. On the other hand, the image of the victim is clear in the mental health literature, primarily addressing poor mental health outcomes, such as post-traumatic stress disorders (PTSD). Although various individual factors have also been identified as risk factors, robust examination of disaster exposure and psychosocial resources (e.g., psychiatric pre-morbidity and personality) seems to be unique to the mental health literature [12-14]. In both literatures, the contributions of several common individual factors such as age, sex, and education are controversial.”

*C3.3) Method:*

*C3.3.1a) Is the data collected from 2011 year or 3600 people who experienced 2011? If the answer is former, why did this study conducted so late? If answer is the latter one, when was this survey conducted?*

*C3.3.1b) How did the author(s) collect the survey questions? By email, by post service, or some 3rd party organization or data source that the author(s) use it for this study?*

R3.3.1) The questionnaire battery was mailed to the participants in early December 2013 and returned by mid-January 2014. We clarified the period and sending methods of the survey in the revised Methods section as follows (L116–122):

“In early December 2013, a questionnaire battery was mailed to 3,600 residents who were randomly sampled from the electoral registers (and thus were aged 20 years or older) of tsunami-affected districts or temporary settlements in the four most populated coastal cities (Ishinomaki, Kesen-numa, Natori, and Sendai) in Miyagi Prefecture, where the damage caused by the earthquake and tsunami was most severe. In total, 1,412 questionnaires (39%) were anonymously completed and returned by mid-January 2014.”

*C3.3.2) Why did the author(s) select 18 categories? Why not 15 or 20?*

R3.3.2) It was not our intention to choose ‘18’ areas or items. We tried to cover as many difficulties as possible based on our own experience and knowledge, and as a result, we ended up with 18 items. We admit, however, that the original phrase "The following 18 items were selected:" makes it sound as if we intentionally chose 18 items. Therefore, we removed this number and modified it as follows (L133–139):

“The areas of difficulty in the aftermath of the earthquake and tsunami were selected to cover as many difficulties as possible based on our own experience and knowledge. The following items were selected: 1) eating, 2) cooking, 3) appropriate spare clothes, 4) room temperature, 5) sleeping, 6) access to a toilet, 7) washing one’s face, 8) bathing, 9) laundry, 10) information gathering, 11) transportation, 12) medical care for oneself, 13) medical care for one’s family, 14) psychological stress, 15) psychological care for one’s family, 16) noise, 17) stench, and 18) privacy.”

*C3.3.3) What is the DV in this study? You observed the 18 categories on what?*

R3.3.3) The dependent variable of our primary analysis was the resolution success of each difficulty type (i.e., obtained from the factor analysis of the 18 difficulty items) separately for the six solution types (self-help, request for help, help from family, help from an acquaintance, help through cooperation, and public assistance). That is, our primary analysis composed of 12 regression analyses with different dependent variables and the same set of explanatory variables. We admit that our original manuscript did not provide sufficiently clear view of the purpose and primary analysis. We believe that they are now clear thorough the clarification of the aim (L87–89; see R3.2.4) and method outline in revised Introduction as follows (L99–103):

“First, we explored the major types of difficulty among the 18 areas using a cluster analysis; characteristics of these difficulty types were examined in terms of frequency, relevant resolution types, and exacerbating factors. Then we explored the individual factors that facilitated the resolution of each difficulty type separately for the six solution types.”

*C3.4) Analysis:*

*C3.4.1) How come the study was performed with hierarchical regression? What are the motives for this? The reader should be prepared for this in earlier section(s).*

R3.4.1) We used hierarchical regression to examine the contribution of personality factors after controlling for the effect of general background (i.e., damage and demographic) factors. It is now explicitly explained in the method outline of the revised Introduction as follows (L103–105):

“To examine contributing individual factors, a hierarchical regression analysis was used. We first identified contributing background factors and then asked whether any personality factor had additional explanatory power.”

*C3.4.2) Some of the tables should be condensed or removed or reorganized. It seems too fluffy by having more than 15 tables! More like a report I would say rather than a scientific paper.*

R3.4.2) We extensively reorganized the tables to improve the readability. Original Table 1 was reformatted to new Table 1 and original Tables 2 and 3 are now in the supplementary (S1 and Tables, respectively). Original Table 4 was divided into the degree of difficulty and resolution success (new Tables 2 and 3, respectively). Original Tables 5-18 are now all in the supplementary (S3-16 Tables, respectively).

*C3.5) Discussion and conclusions*

*C3.5.1) Same comment applies for the discussion. Just discuss the main findings. It seems too long.*

R3.5.1) Discussion was thoroughly reorganized and rewritten to improve the clarity. Particularly, three long paragraphs previously dedicated to extended (e.g., anthropological) discussion on each personality factor identified were now totally removed. Together with the clarification of the research purpose, we focused on our main finding and believe the revised Discussion is now much more readable. As a result, the length of our Discussion was drastically diminished (from 2,896 to 1,553 words).

*C3.5.2) On the other hand, conclusion should be highlighted as it is the most important part of this study.d..*

R3.5.2) Conclusions section was totally rewritten to highlight our most important findings as follows (L488–514):

“Analyzing the data from the 2011 Great East Japan Earthquake and Tsunami Disaster, we explored individual background and personality factors that facilitate self-aid or mutual assistance in the immediate aftermath of a disaster considering different types of difficulty and resolution. We first identified two types of difficulties: general life difficulties, which were experienced by most victims, and medico-psychological difficulties, which were experienced to varying degrees across victims. Both types were exacerbated by disaster damages, whereas medico-psychological difficulties were uniquely affected by personality factors, with neuroticism and extraversion acting as exacerbating and mitigating factors, respectively.

Then we examined the effect of individual factors on success in resolving the two types of difficulty through six types of resolution: self-resolution, four types of mutual assistance, and public assistance. In general, disaster damage hampered self-resolution and forced a reliance on cooperation or public assistance. Some demographic factors, such as being young and living in a three-generation household, facilitated resolution thorough the family. Importantly, several personality factors facilitated different types of resolution, primarily of general life difficulties: problem solving facilitated self-resolution, altruism or stubbornness facilitated resolution through request, leadership facilitated resolution through acquaintances, and emotion regulation facilitated resolution through public assistance.

Our results demonstrate the importance of dissociating difficulties and resolution types when identifying facilitative factors for self-aid and mutual assistance in this disaster stage. The identified involvement of different individual factors, particularly personality factors, in different types of resolution may reflect the complexity of social dynamics as well as the psychological processes involved in survival in this context. The current findings contribute to the improvement of disaster risk mitigation through risk evaluation and the fostering of resilience among citizens by allowing separate consideration of different difficulty types and resolution processes.”
